# PACT is requisite for prostate cancer cell proliferation

**DOI:** 10.1038/s41598-025-20494-9

**Published:** 2025-10-21

**Authors:** Dianne J. Beveridge, Andrew J. Woo, Kirsty L. Richardson, Rikki A. M. Brown, Lisa M. Stuart, Manjot Singh, Andrew D. Redfern, Peter J. Leedman

**Affiliations:** 1 Laboratory for Cancer Medicine, Harry Perkins Institute of Medical Research, and Centre for Medical Research, The University of Western Australia, Crawley, WA 6009 Australia; 2 Centre for Precision Health, Edith Cowan University, Joondalup, WA 6027 Australia; 3 School of Medicine and Pharmacology, The University of Western Australia, Crawley, WA 6009 Australia; 4 Department of Medical Oncology, Fiona Stanley Hospital, Murdoch, WA 6150 Australia

**Keywords:** Prostate cancer, Proliferation, PACT, PSA, Cell-cycle, Targeted therapies, Prostate cancer

## Abstract

PACT (encoded by the *PRKRA* gene) is a double-stranded RNA binding protein with defined antiviral defense and cytoplasmic RNA-induced silencing actions in mammals. We previously described a further role for PACT as a modulator of nuclear receptor (NR)-regulated gene expression. Here, we investigated the role of PACT in prostate cancer (PCa) using a loss-of-function approach. Depletion of PACT in multiple PCa cell lines resulted in a reduction in cell proliferation, but viability was maintained. RNA-sequencing analysis of LNCaP PCa cells ± PACT revealed a depletion of biological processes involved in cell cycle, mitochondrial function, and NR-response pathways in the PACT knockout (KO) cells. In the PACT KO cells, downregulated genes included the androgen-regulated *KLK3* (prostate specific antigen, PSA), together with *H2AFJ, PSMD5, AQP3, TMEM45B*, and *SLC22A3,* and siRNA-mediated knockdown of these genes reduced cell growth and proliferation in LNCaP cells. Further, reducing PACT or PSA induced cell cycle arrest at G0/G1. Additionally, the hormone-mediated upregulation and AR antagonist-driven downregulation of PSA gene expression were respectively attenuated and enhanced in PACT KO cells. Taken together, these data support a pro-proliferative role for PACT in PCa, and siRNA therapeutic targeting of PACT, or downregulated genes with PACT KO, could represent a new therapeutic approach.

**Supplementary Information:**

The online version contains supplementary material available at 10.1038/s41598-025-20494-9.

## Introduction

Prostate cancer (PCa) is the fourth most commonly diagnosed cancer worldwide, and in 2020 accounted for 3.8% cancer related death in men^1^. The increasing awareness of PCa, coupled with improved radiological detection methods and the evolution of Prostate Specific Antigen (PSA)-based screening, has contributed to an escalation in PCa diagnoses in recent years^2^. However, due to early diagnosis the majority of PCa patients have localized, low-risk disease, and have a good prognosis with close monitoring and locally directed therapy as required^3^. However, a proportion of men experience disease recurrence after local therapy or present with de novo metastatic disease. At metastatic disease onset, PCa tumor growth is predominantly androgen-dependent, laying the foundation for the development of therapies targeting the androgen receptor (AR), including the anti-androgens abiraterone acetate (inhibits androgen biosynthesis), enzalutamide (MDV3100, interferes with AR signaling)^4^, and apalutamide (ARN-509, prohibits AR signaling)^5^. Inevitably, the vast majority of PCa tumors become resistant to these drugs, and the disease is now termed castrate resistant PCa (CRPC)^4–7^. Current chemotherapies for the treatment of CRPC predominantly target microtubules (e.g., docetaxel, cabazitaxel), and although they have modest efficacy at the castrate-sensitive stage, drug resistance remains almost inevitable^8,9^. Immunotherapy (e.g., sipuleucel-T), and management of bone metastasis with bone-targeted therapy (e.g., radium-233), have also provided modest survival benefits for patients with CRPC^9–12^. Recent advances in systemic therapies for the effective treatment of CRPC include targeting the highly expressed prostate specific membrane antigen (PSMA) with radioligand therapy i.e., ^177^Lu-PMSA-617, polyadenosine diphosphate-ribosome polymerase inhibitors (PARPi) e.g., talazoparib^12–14^, and ultrasound therapy, particularly when combined with anticancer drugs contained within or on ‘microbubbles’ or targeted nanobubbles to PSMA for targeted drug delivery and release^15–18^. Furthermore, siRNA-based therapies for PCa have been the focus of increasing investigation, targeting a wide range of genes (e.g., MDM2, IGHG1, VEGF), and tumor-targeted delivery explored using vehicles such as nanoparticles and dendrimers, yielding varying degrees of success^19^. Despite these advances in treatment options for CRPC, the disease remains incurable, emphasizing the unmet clinical need for new PCa therapeutics.

PACT was initially discovered as a facilitator of mammalian anti-viral defense mechanisms by activating the double-stranded RNA-activated protein kinase (PKR)^20^. In addition, PACT activates retinoic acid-induced gene 1 (RIG-1) mediated signaling to regulate interferon induction following viral infection^21,22^. Further, several studies support a requisite role for PACT in the facilitation of cell growth in non-cancerous scenarios, including in ear development and hearing, in germline stem cell fate, in postnatal anterior pituitary proliferation, and in skull and brain development^23–27^. Subsequently, PACT, together with transactivation response RNA binding protein (TRBP) and Dicer, has been described as an integral member of the cytoplasmic RNA-induced silencing complex (RISC), which regulates the processing of microRNAs for the targeted silencing of gene expression through mRNA cleavage, or translational repression^28,29^.

We previously reported PACT to also be a nuclear receptor (NR)-coactivator, which regulated AR activity and downstream gene expression in PCa, providing linkages between NR-coregulators and the microRNA processing machinery^30^. PACT overexpression promoted tumorigenesis in other cancers, including epithelial skin cancer, hepatocellular carcinoma, colorectal adenocarcinoma, and pancreatic cancer^31–34^, and attributed to chemoresistance in mucinous ovarian and basal-like breast cancers^35,36^.

In the present study, we investigated the role of PACT in PCa using a loss-of-function approach, to further understand its potential as a prospective PCa therapeutic target. Depletion of PACT from human PCa cell lines resulted in a substantial reduction in cell proliferation, cell cycle arrest at G0/G1, and an increase in apoptosis. RNA-sequencing analysis of differentially expressed genes between LNCaP parental and LNCaP PACT knockout cells, revealed a reduction in biological processes and hallmark gene sets pertaining to cell proliferation when PACT was depleted. Small interfering RNA (siRNA) mediated targeting of several downregulated genes; including *H2AFJ, PSMD5, AQP3, TMEM45B*, *SLC22A3,* and *KLK3* (PSA), in LNCaP parental cells recapitulated the functional effects of PACT knockdown. Further, in PACT KO cells the androgen-mediated increase or the AR antagonist-mediated decrease in PSA expression were attenuated or augmented, respectively. These data collectively support the notion that PACT is proproliferative in PCa, and the potential to therapeutically target PACT, or the genes that are downregulated in the absence of PACT, could be of benefit to overall PCa patient survival.

## Results

### PACT sustains proliferation in prostate cancer cells

**Fig. 1:**
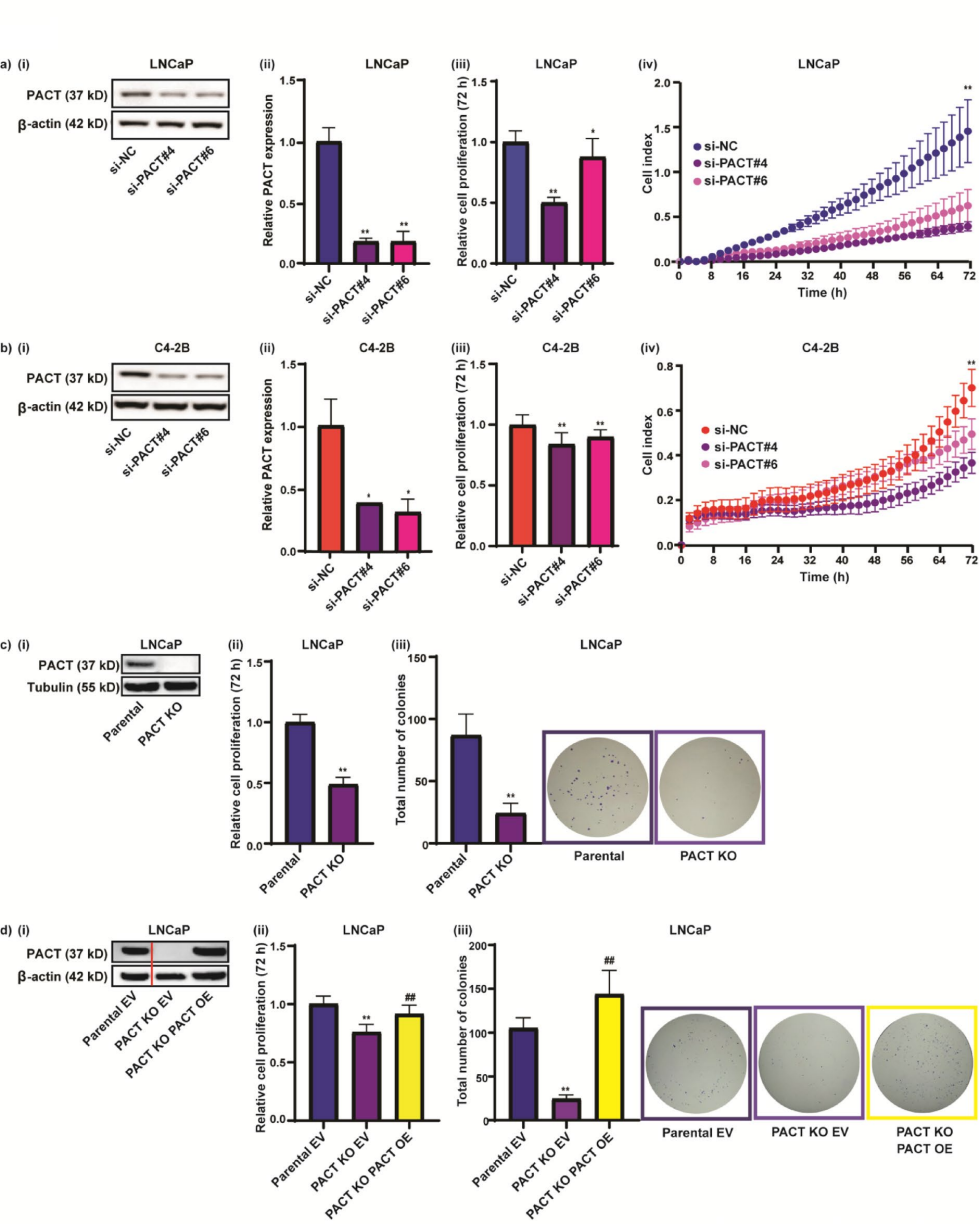
PACT sustains proliferation in prostate cancer cells. (**A**) LNCaP or (**B**) C4-2B PCa cells were treated with negative control siRNA (si-NC) or two different PACT siRNAs (si-PACT#4 and si-PACT#6) for one day and then harvested for RNA extraction or for seeding into appropriate plates for and various times for (**i**) western blot for PACT expression, β-actin loading control, 72 h, see Supplementary Fig. S6A-B for original blots; (**ii**) RT-qPCR validation of si-PACT knockdown at 1 d post-transfection. Expression of PACT mRNA is normalised to GAPDH housekeeping gene expression, calculated using the 2^-ΔΔCt^ method, and relative to si-NC; (**iii**) cell titre proliferation assay (72 h); and (**iv**) xCELLigence assay (72 h). (**C**) Comparison of LNCaP parental and CRISPR PACT knockout (PACT KO) cells at various times post-plating of equal number of cells (**i**) western blot for PACT expression, tubulin loading control, 72 h, see Supplementary Fig. S6C for original blot; (**ii**) cell titre proliferation assay (72 h); and (**iii**) colony forming assays (~ 2 weeks). (**D**) Comparison of LNCaP parental and PACT KO cells stably overexpressing empty vector (EV) or PACT cDNA (PACT OE) at various time points post-plating of equal number of cells (**i**) western blot for PACT expression, β-actin loading control, 72 h, red line indicates non-adjacent lanes from western blot used for figure (see Supplementary Figure S6D); (**ii**) cell titre proliferation assay (72 h); and (**iii**) colony forming assays (~ 2 weeks). Error bars = SD (or SE for RT-qPCR) and are representative of three independent experiments. Data analysis was with an unpaired two-tailed student’s t-test; with significance denoted as: *p < 0.05, **p < 0.005 relative to si-NC or parental cells; or ^##^*p* < 0.005 PACT KO PACT OE relative to PACT KO EV.

To identify the functional effects of PACT expression on PCa cell growth we performed loss-of-function studies in multiple human PCa cell lines. We initially used siRNA to mediate the transient knockdown of PACT in the androgen-responsive LNCaP and C4-2B cells using two different PACT siRNAs (IDs s16334 and s16336; hereafter si-PACT#4 and si-PACT#6). We observed a greater than 70% knockdown of PACT at both the protein and RNA level in both cell lines (Fig. 1A(i–ii), B(i–ii). We subsequently assessed the impact of the siRNA mediated reduction of PACT had on cell proliferation using a Cell Titer end-point assay and the xCELLigence real time system, and the cells with less PACT exhibited a reduction in proliferation as compared to the negative control (si-NC) transfected cells using both methods of evaluation (Fig. 1A(iii–iv), B(iii–iv)). A similar result was observed when we transiently transfected siPACT#4 into the androgen-responsive 22Rv1 and VCaP cell lines; and the androgen-independent PC3 and DU145 cell lines (Supplementary Fig. S1A-D). We next explored if the reduced growth phenotypes observed in the siPACT treated PC3 and DU145 cells was associated with the regulation of the AR-coactivators implicated in enzalutamide resistance, namely GATA-binding protein 2 (GATA2) and growth regulating estrogen receptor binding 1 (GREB1)^37^, and observed no significant differences in the regulation of these genes in both cell lines with PACT depletion (Supplementary Fig. S1E (i-ii)). Taken together, these data indicate a requisite role for PACT in PCa cell proliferation, and this role is in part not dependent on AR or its co-regulators.

As an independent approach to deplete PACT, we used the CRISPR-Cas9 genome editing technique to knock PACT out of the androgen-responsive LNCaP cells. PCR and sequencing confirmed correct targeting of CRISPR-Cas9 to the PACT locus (Supplementary Fig. S1F(i-iii)), and PACT protein depletion verified by western blot (Fig. 1C(i)). There was a considerable reduction in the proliferation of the PACT knockout (KO) cells compared to the parental cells as assessed by Cell Titer assay (Fig. 1C(ii)), which was in concordance with the PACT siRNA knockdown data (Fig. 1A). Furthermore, colony formation assays showed that LNCaP parental cells formed more colonies than their PACT KO counterparts (Fig. 1C(iii)), further supporting a requirement for PACT in cell proliferation, albeit dispensable for cell survival.

To investigate if the reduced cell proliferation in PACT KO cells is directly due to the absence of PACT, we used lentiviral transduction of PACT cDNA to reconstitute PACT protein in the KO cells (confirmed by western blotting, Fig. 1D(i)). Re-constitution of PACT via ectopic cDNA expression in the PACT KO cells restored cell proliferation back to the level observed in LNCaP parental cells in Cell Titer and colony formation assays (Fig. 1D(ii–iii)). The loss-of-function studies using siRNA and CRISPR KO, together with the rescue experiment, suggest a direct role for PACT in sustaining PCa cells proliferation.

### PACT expression positively correlates with genes involved in metabolic processes

Based on the loss-of-function studies using established PCa cell lines, PACT is required for sustaining the proliferation of PCa cells and to investigate if this association with proliferation also exists in clinical samples, we interrogated the Prostate Adenocarcinoma samples within the TCGA PanCancer Atlas (n = 494). Using the Database for Annotation, Visualization and Integrated Discovery (DAVID) we performed gene ontology (GO) pathway analyses on the top 200 genes which showed positive or negative correlation to *PRKRA/*PACT expression (see gene lists in Supplementary Tables 1 and 2). The biological processes of topmost co-expressed genes included those involved with mitochondrial function and associated metabolic pathways, with ATP5PB, a gene which encodes a subunit of mitochondrial ATP synthase, being one of the most highly correlated genes (Supplementary Fig. S2A (i-ii)). In contrast, the genes that are inversely correlated to PACT are enriched with biological processes involving epigenetic regulation and chromatin organization, including the ASXL1 gene, which has been reported to be a tumour suppressor (Supplementary Fig. S2B (i-ii) and Wu et al.^38^). Based on the requirement for PACT in PCa cell proliferation, and the co-expressed gene pathways in patients, we hypothesize that functionally PACT enhances cell growth by regulating high energy requiring metabolic processes.

### Targeted knockdown of genes downregulated in the absence of PACT affects PCa cell proliferation

**Fig. 2:**
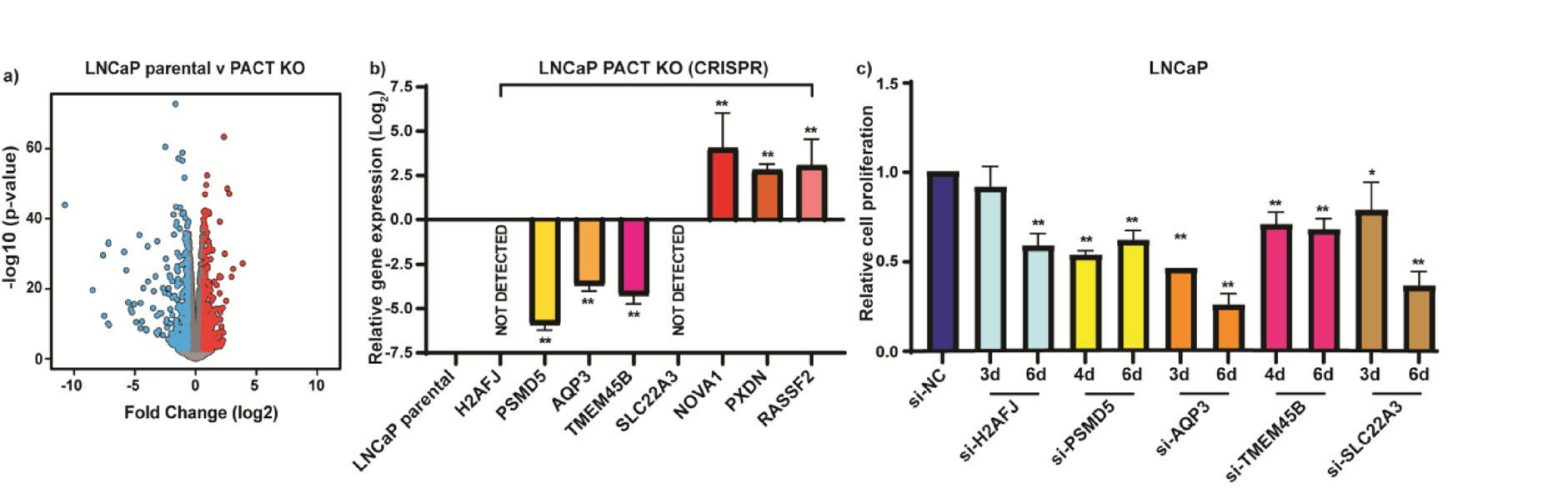
Targeted knockdown of genes downregulated in the absence of PACT affects PCa cell proliferation. RNA-sequencing (RNA-seq) analysis was performed on LNCaP parental vs LNCaP PACT CRISPR knockout (PACT KO) cells. (**A**) Volcano plot of the differentially expressed genes between the two cell lines, using ≥ 1.5 absolute fold change (FC) (0.58 log_2_ FC) and a False Discovery Rate (FDR) of *p* < 0.01, with blue dots representing genes downregulated, and red dots representing genes upregulated with PACT KO. (**B**) RT-qPCR validation of differentially expressed genes in LNCaP parental and LNCaP PACT KO cells. Expression of mRNAs is normalised to HPRT housekeeping gene expression, calculated using the 2^-ΔΔCt^ method, and relative to parental LNCaP. Error bars = SE; n = 3; data analyses used an unpaired two-tailed student’s t-test; with significance denoted as: **p < 0.005 relative to parental cells. (**C**) Transient transfection of LNCaP cells was with 20 nM gene specific siRNAs or si-NC and the effects of gene knockdown measured via cell proliferation assay at 2- to 6-days post-transfection. See Supplementary Fig. S3B for validation of siRNA mediated gene knockdown. Error bars = SD; n = 3; data analysis was with an unpaired two-tailed student’s t-test; with significance denoted as: *p < 0.05, **p < 0.005 relative to si-NC.

To explore the impact of PACT depletion on gene expression in PCa cells, we performed RNA-Sequencing (RNA-seq) analysis of LNCaP parental versus PACT CRISPR KO cells. Using ≥ 1.5 absolute fold change (FC) (0.58 log_2_ FC) and a False Discovery Rate (FDR) of *p* < 0.01, we identified 718 differentially expressed genes (DEGs) between the two groups; 343 DEGs were downregulated, and 375 DEGs were upregulated in the absence of PACT (Fig. 2A and Supplementary Tables 3 and 4).

We validated five of the most downregulated and three of the most upregulated genes in the PACT KO cells from the RNA-seq. *H2AFJ* (H2A histone family member J)*, PSMD5* (proteasome 26 s non-ATPase subunit, 5)*, AQP3* (Aquaporin 3), *TMEM45B* (transmembrane protein 45B), and *SLC22A3* (Solute carrier family 22 member 3) were respectively downregulated 10.76, 7.18, 5.88, 4.63, and 3.9 log_2_ fold; and *NOVA1* (neuro-oncological ventral antigen-1), *PXDN* (Peroxidasin), and *RASSF2* (Ras association domain-containing protein 2), were respectively upregulated 2.77, 2.33, and 1.4 log_2_ fold, in PACT KO cells (Supplementary Tables 3 and 4). RT-qPCR quantification of each of these genes in LNCaP parental versus PACT KO cells verified the RNA-seq data (Fig. 2B).

We next used siRNA to transiently knockdown *H2AFJ*, *PSMD5*, *AQP3*, *TMEM45B*, and *SLC22A3* gene expression in LNCaP PCa cells, to assess the functional effects of these molecules on PCa growth. We initially evaluated three or four different siRNAs (20 nM) for each gene (Supplementary Fig. S3A (i-v)) and selected optimal siRNAs for our experiments (#s31462 *H2AFJ*; #SI03167108 *PSMD5*; #s1521 *AQP3*; #s42359 *TMEM45B*; and #s13107 *SLC22A3*). We transiently transfected LNCaP cells with either gene-specific siRNA or si-NC (20 nM) and assessed cell proliferation using a Cell Titer assay at 2 to 6 days post-transfection. Gene-specific siRNA transfected cells exhibited a substantial growth reduction as compared to the si-NC transfected cells for each gene (Fig. 2C), supporting the concept that downregulating PACT, or the genes which are downregulated in the absence of PACT could favorably impact clinical prostate cancer progression.

### PACT regulates PCa cell cycle progression

**Fig. 3:**
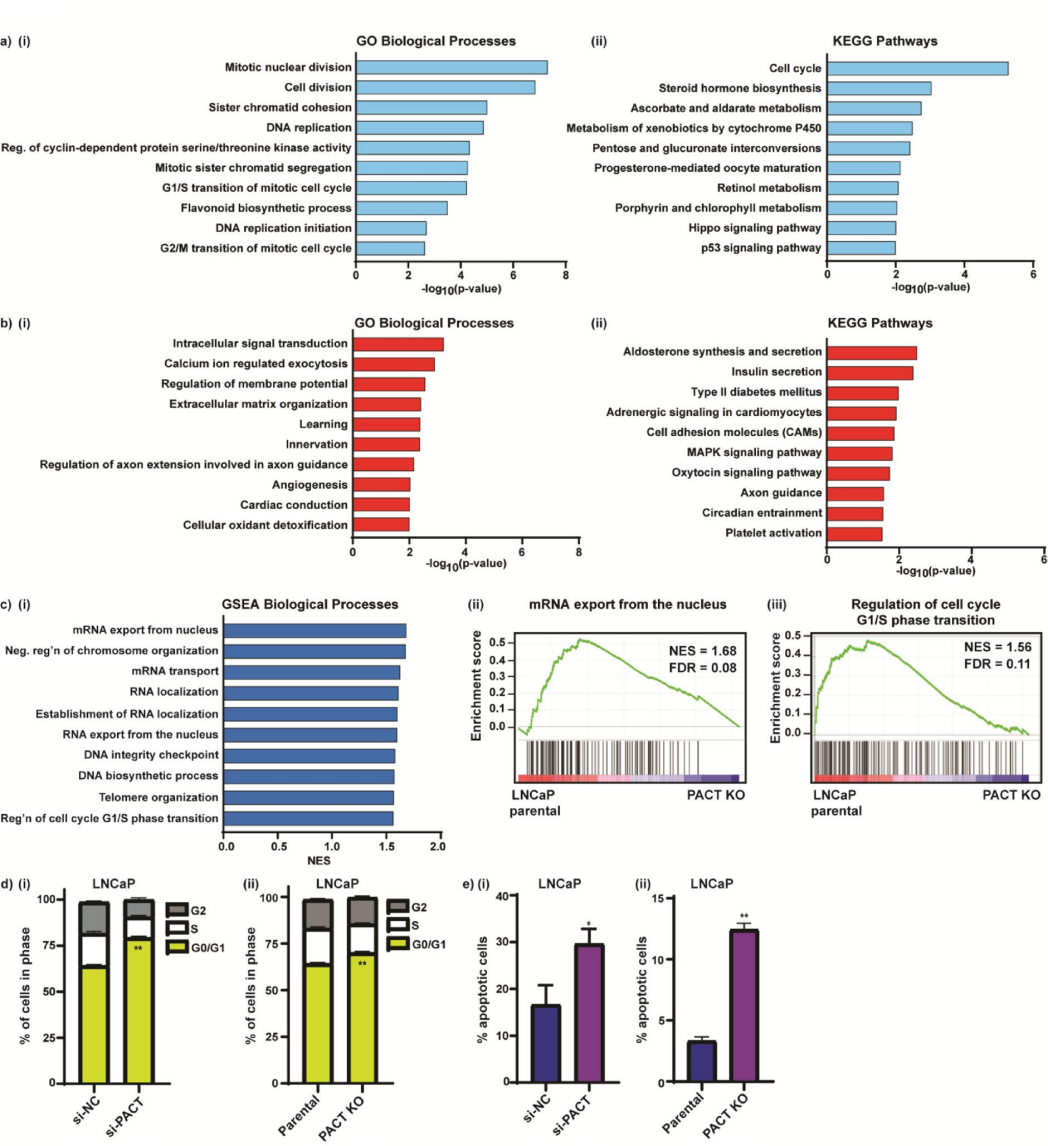
PACT regulates PCa cell cycle progression. (**A**, **B**) DAVID gene ontology (GO) enrichment analyses of the pathways downregulated (**A**) and upregulated (**B**) with PACT KO; (**i**) GO Biological processes, and (**ii**) KEGG pathways. (**C**) Gene Set Enrichment Analysis (GSEA) of the RNA-seq (**i**) biological processes; and (**ii**–**iii**) representative enrichment score plots. The y-axis and the green line show the enrichment score for each gene, illustrated as a vertical line plotted in rank order of the most gene abundance (red, left) to the least gene abundance (blue, right) within the indicated samples (as log_2_FC/comparison); the black vertical lines correspond to member genes from the set. NES = normalized enrichment score, FDR = false discovery rate. Pathways ranked by the *p*-value result of a Fisher’s exact test. (**D**–**E**) Flow cytometry cell cycle analysis (**D**) and apoptosis analysis (**E**) of LNCaP cells ± PACT; (**i**) LNCaP cells transfected with si-NC or si-PACT (20 nM) for 72 h, and (**ii**) LNCaP parental and LNCaP PACT KO cells assayed 72 h post-plating. n = 3; data analysis was with an unpaired two-tailed student’s t-test; with significance denoted as: *p < 0.05, **p < 0.005 relative to si-NC or parental cells. See Supplementary Fig. S3C (i-ii) for RT-qPCR validation of si-PACT knockdown.

We next interrogated the RNA-seq gene expression data using DAVID GO and Kyoto Encyclopedia of Genes and Genomes (KEGG)^39–41^ pathway analyses of the downregulated DEGs and identified mitotic nuclear division, cell division, cell cycle, and steroid hormone biosynthesis as the most enriched biological processes (Fig. 3A(i–ii)). The pathway analyses of the upregulated genes were mixed and less significant than the downregulated processes, without clear mechanistic trends (Fig. 3B(i–ii)). Further, Gene Set Enrichment Analysis (GSEA) of the RNA-seq data (FDR < 0.25, *p* < 0.01) showed a depletion of gene sets and biological processes relating to cell cycle and proliferation, and to RNA transport and localization in the KO cells e.g., regulation of cell cycle G1/S phase transition, and mRNA export from the nucleus (Fig. 3C(i–iii)). Moreover, biological processes relating to chromosome organization and DNA integrity and biosynthesis were also depleted in the PACT KO cells (Fig. 3C(i)), congruent with the reported roles of PACT, that is acting via PKR and the RISC complex^20,28^.

The enrichment of pathways relating to cell division in the downregulated DEGs in the PACT KO cells corroborated the reduced proliferation phenotype observed in the PACT loss-of-function studies. To confirm the finding, we performed cell cycle analyses in LNCaP PCa cells with either transient (siRNA) or stable (CRISPR) mediated PACT depletion. Using propidium iodide (PI) staining and flow cytometry we detected an increase in cell cycle arrest at G0/G1 and a block in transition to S phase using both methods of PACT knockdown (Fig. 3D(i–ii)). Due to the cell cycle arrest at G0/G1, we also investigated the effect of PACT reduction on apoptosis in LNCaP cells using annexin V/PI staining and flow cytometry and observed an increase in apoptotic cells with PACT depletion (Fig. 3E(i–ii)). Taken together, these data support the notion that the growth reduction in PACT KO cells is, in part, via alteration of cell cycle progression and apoptosis, further validating the proproliferative function of PACT in PCa growth.

### PACT modulates key androgen receptor signaling molecules

**Fig. 4:**
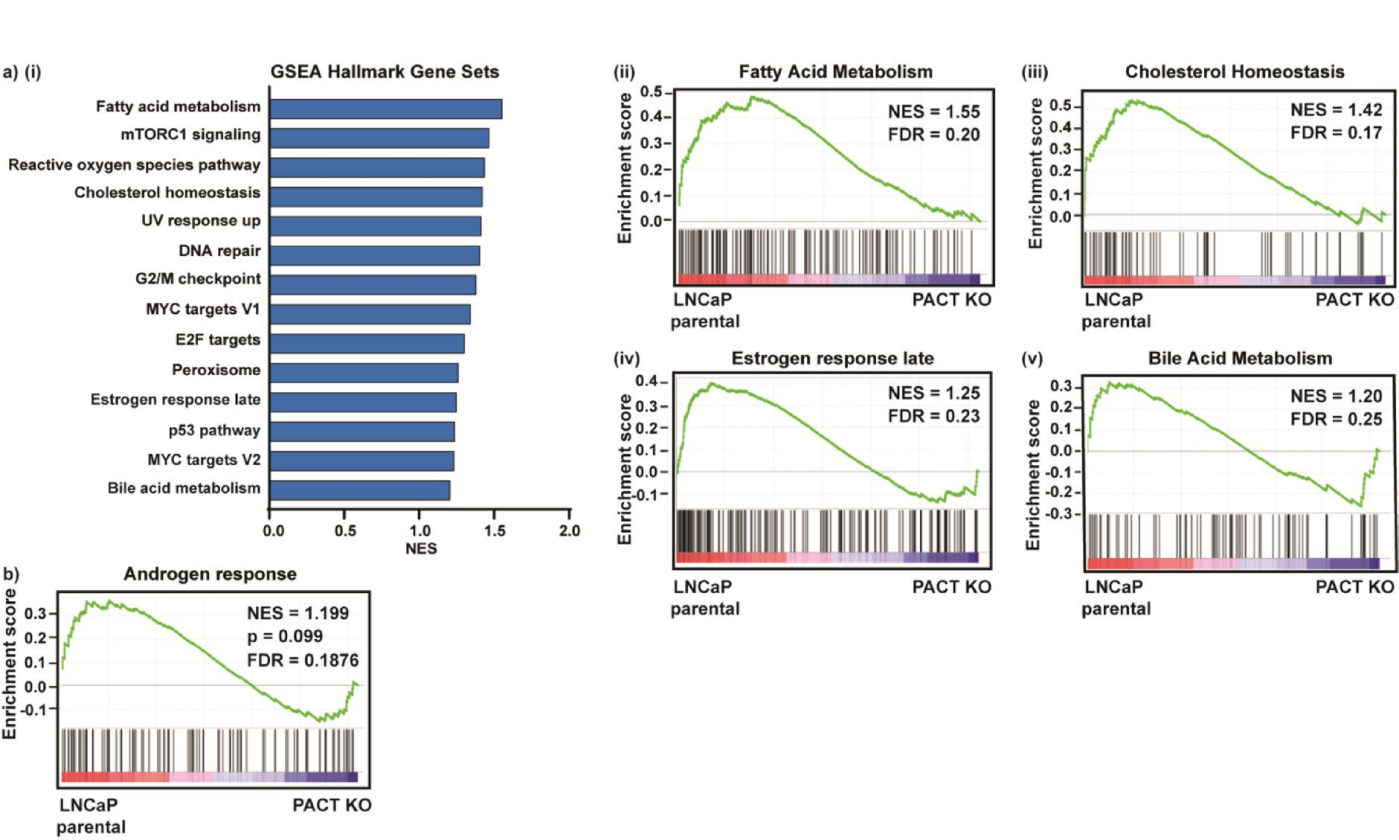
PACT modulates key androgen receptor signaling molecules. (**A**, **B**) GSEA and representative enrichment score plots of the RNA-seq. (**A**) Hallmark gene sets, and (**B**) Androgen response Hallmark. The y-axis and the green line show the enrichment score for each gene, illustrated as a vertical line plotted in rank order of the most gene abundance (red, left) to the least gene abundance (blue, right) within the indicated samples (as log_2_FC/comparison); the black vertical lines correspond to member genes from the set. NES = normalized enrichment score, FDR = false discovery rate. Pathways ranked by the *p*-value result of a Fisher’s exact test.

Interestingly, some of the depleted GSEA Hallmark gene sets in the PACT KO cells were NR-function related, including fatty acid metabolism, cholesterol homeostasis, estrogen response late, and bile acid metabolism (Fig. 4A(i–v)). As we previously described PACT as a NR-coregulator regulating AR activity and downstream gene expression in PCa, we performed GSEA against Hallmark for Androgen response. We observed the upregulation of Androgen response genes in the parental cells relative to the PACT KO cells, indicating a depletion of the gene set in the absence of PACT (Fig. 4B).

### PACT levels modify the expression of PSA in response to androgens and anti-androgens in PCa cells

**Fig. 5:**
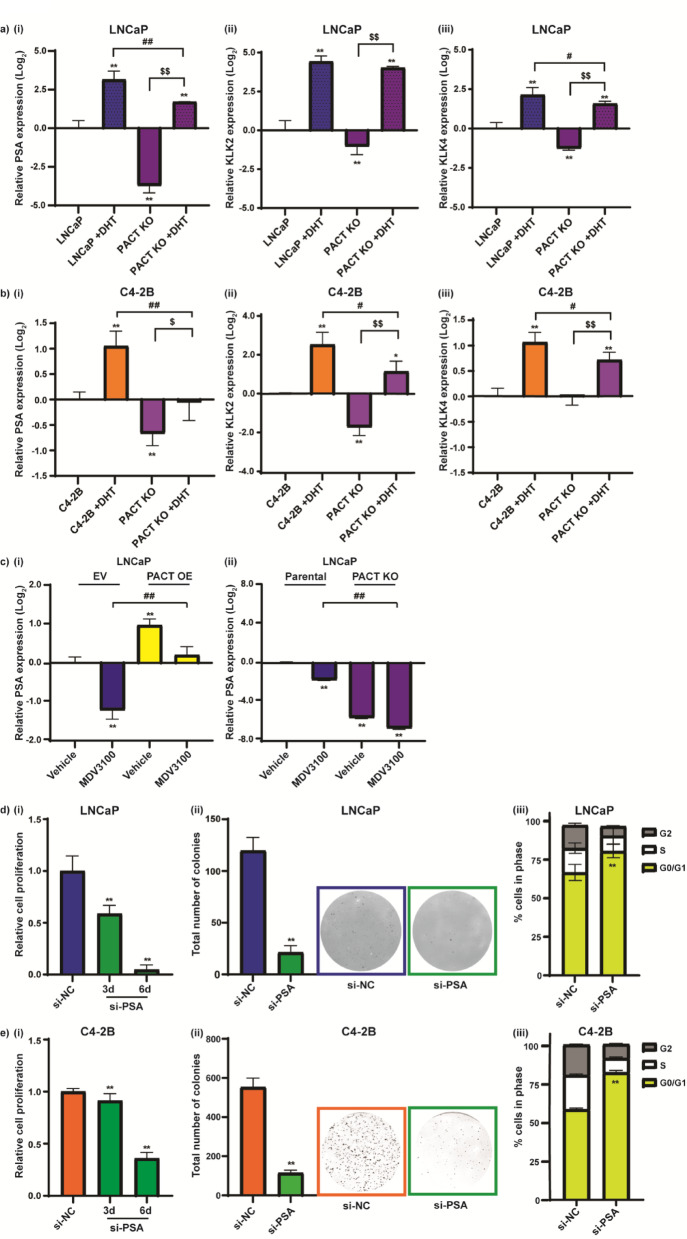
PACT levels modify the expression of PSA in response to androgens and anti-androgens in PCa cells, and PSA regulates PCa cell proliferation and cell cycle progression. (**A**) LNCaP or (**B**) C4-2B parental and PACT KO cells were treated with ± 10 nM DHT for 24 h, and RNA harvested for RT-qPCR analysis for: (**i**) PSA mRNA expression (**ii**) KLK2 mRNA expression, and (**iii**) KLK4 mRNA expression. (**C**) LNCaP cells stably overexpressing Empty Vector (EV) or PACT (PACT OE) (**i**); and LNCaP parental and PACT knockout (PACT KO) (**ii**) were treated with ± 1 uM MDV3100 for 24 h, and RNA harvested for RT-qPCR analysis of PSA mRNA expression. (**D**, **E**) LNCaP or C4-2B cells were transiently transfected with 20 nM PSA siRNA (si-PSA) or negative control siRNA (si-NC) and assayed for the effects of gene knockdown via (**i**) cell proliferation at 3 d and 6 d post-transfection; (**ii**) colony formation at ~ 2 weeks post-transfection; and (**iii**) flow cytometry cell cycle analysis 72 h post-transfection. See Supplementary Fig. S4 (**E**, **F**) for validation of siRNA mediated PSA knockdown. Expression of mRNAs via RT-qPCR is normalised to GAPDH or HPRT housekeeping gene expression, calculated using the 2^-ΔΔCt^ method, and relative to parental LNCaP (no treatment); error bars = SE; n = 3; data analyses used an unpaired two-tailed student’s t-test; with significance denoted as: ns (non-significant) *p* > 0.05; **p < 0.005 relative to parental cells (vehicle); ^#^*p* < 0.05 and ^##^*p* < 0.005 LNCaP + DHT/MDV3100 relative to PACT OE/KO + DHT/MDV3100; ^$$^*p* < 0.005 PACT KO relative to PACT KO + DHT. For cell proliferation, colony forming assays, and cell cycle assays; error bars = SD; n = 3; data analyses used an unpaired two-tailed student’s t-test; with significance denoted as: **p < 0.005 relative to si-NC.

We further scrutinized the RNA-seq data to determine if known androgen-regulated genes were differentially expressed in the PACT KO cells, and notably the Kallikrein family members *KLK3* (Kallikrein related peptidase 3, Prostate-specific antigen, PSA), *KLK2* (Human kallikrein), and *KLK4* (PSA-related serine protease) were all downregulated (2.49, 0.83 and 0.47 log_2_ fold change, respectively) in the PACT KO cells (Supplementary Table 3, and GSE253245). We treated LNCaP parental and PACT KO cells ± dihydrotestosterone (DHT) for 24 h and validated the differential expression, and hormone responsiveness of these androgen-regulated genes via RT-qPCR and western blotting (PSA), with the PACT KO cells exhibiting both a lower basal and hormone mediated expression of each gene (Fig. 5A (i-iii) and Supplementary Fig. S4A). To further substantiate these findings in an additional androgen-responsive PCa cell line, we used CRISPR-Cas9 to knock PACT out of C4-2B cells (Supplementary Fig. S4B (i)) and treated both C4-2B parental and C4-2B PACT KO cells ± 10 nM DHT or 10 nM of the synthetic androgen metribolone (R1881) for 24 h. The differential expression and hormone responsiveness of PSA and *KLK2* in the C4-2B cells was comparable to observations in LNCaP cells, however *KLK4* expression was not affected by PACT KO (Fig. 5B (i-iii) and Supplementary Fig S4B (ii)).

We next investigated if PACT expression levels in PCa cells could impact the effects of the AR antagonist MDV3100 (enzalutamide). To enable this study, we stably overexpressed PACT in LNCaP cells (PACT OE) (verification of OE see Supplementary Fig. S4C) and treated both these cells and the PACT CRISPR KO cells with 1 uM MDV3100 for 24 h. In the PACT overexpressing cells, the expression of PSA was elevated and the effect of MDV3100 treatment nullified (Fig. 5C (i)), which contrasted with the PACT KO cells, whereby the expression of PSA following MDV3100 was decreased (Fig. 5C (ii)).

### PSA regulates PCa cell proliferation and cell cycle progression

We subsequently used siRNA to transiently knockdown PSA in PCa cells to assess the functional effects of PSA gene expression on PCa cell growth. We initially tested three different PSA targeting siRNAs (Supplementary Fig. S4D) in LNCaP cells and selected #SI03078299 for our studies. siRNA-mediated knockdown of PSA in LNCaP cells resulted in a reduction in cell proliferation at 3- and 6-days post-transfection via a Cell Titer assay, reduced colony formation in clonogenicity assays, and induced cell cycle arrest at G0/G1 (Fig. 5D(i–iii)). Similar results were observed in C4-2B (Fig. 5E(i–iii)) and to a lesser extent in 22Rv1 (Supplementary Fig. S4G (i-iii)) prostate cancer cell lines, both of which express PSA at higher and lower levels to LNCaP cells, respectively (Supplementary Fig. S4H).

Collectively, this data supports the role of PACT in modulating key AR-signaling molecules, the targeting of which could abrogate the proproliferative function of PACT.

### TRBP has a compensatory role for cell survival when PACT is depleted from LNCaP PCa cells

**Fig. 6:**
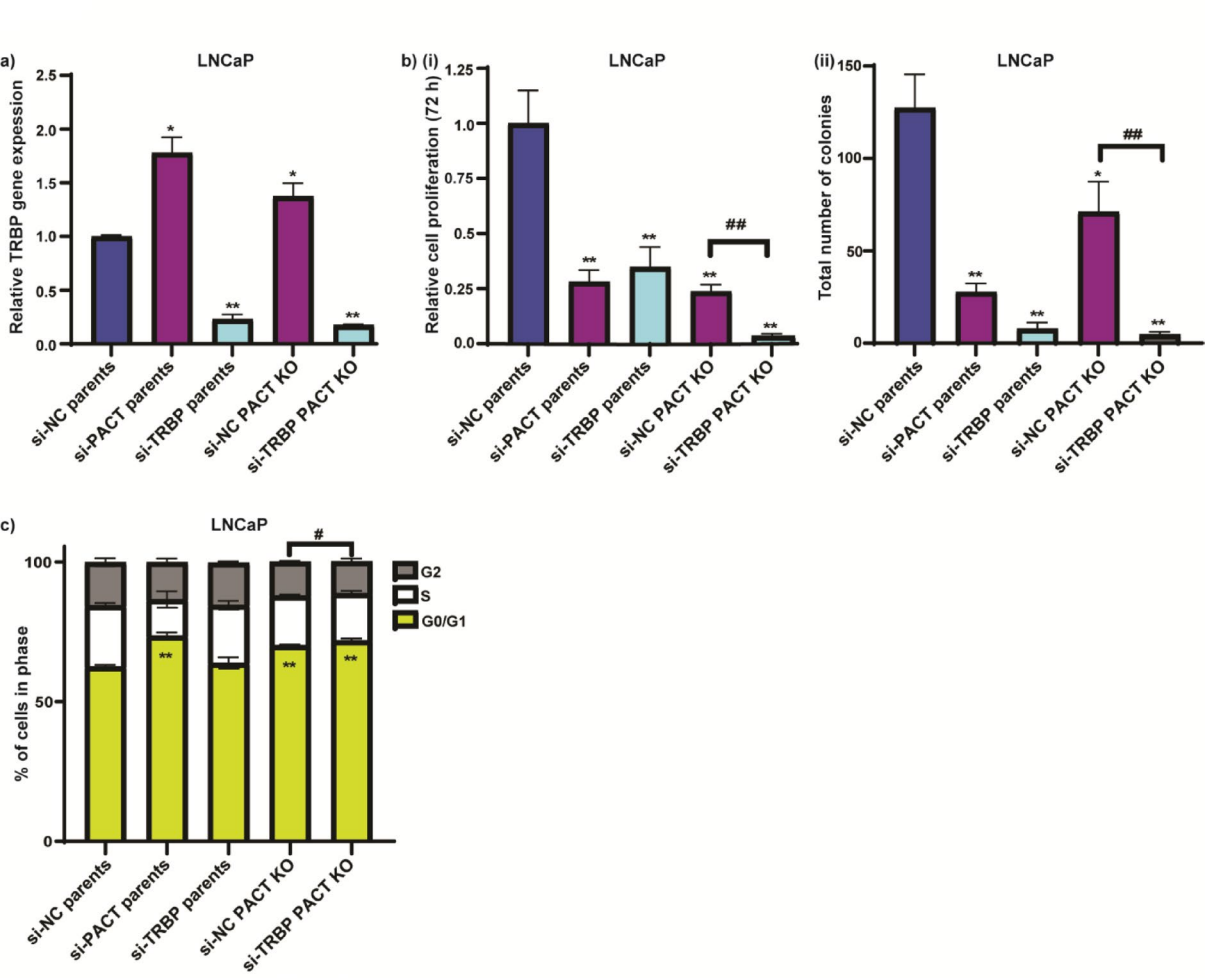
TRBP has a compensatory role for cell survival when PACT is depleted from LNCaP PCa cells. LNCaP parental or PACT KO cells were transiently transfected with either si-NC, si-PACT or si-TRBP at 20 nM, and subsequently assayed for (**A**) TRBP mRNA expression at 72 h post-transfection using RT-qPCR; (**B**) (**i**) cell viability with cell titre assay at 72 h post-transfection; (**ii**) colony formation at ~ 2 weeks post-transfection, and (**C**) flow cytometry cell cycle analysis at 72 h post-transfection. See Supplementary Fig. S5B for validation of siRNA mediated PACT or TRBP knockdown. Expression of TRBP or PACT mRNA is normalised to GAPDH housekeeping gene expression, calculated using the 2^-ΔΔCt^ method, and relative to si-NC parental cells. Error bars = SE (**A**) or SD (**B**, **C**); n = 3; data analyses used an unpaired two-tailed student’s t-test; with significance denoted as: *p < 0.05, **p < 0.005 relative to si-NC parental cells; ^#^*p* < 0.05, ^##^*p* < 0.005 si-NC PACT KO relative to si-TRBP PACT KO cells.

Our findings suggest that PACT sustains PCa cell proliferation, although it is dispensable for cell survival. Given that PACT and TRBP are both integral members of the RISC complex and co-modulate PKR activation, we next used si-TRBP to investigate the role of TRBP in LNCaP cells ± PACT. We first evaluated three different siRNAs targeting TRBP (Supplementary Fig. S5A) and selected #s13790 to use in subsequent experiments. We transiently transfected LNCaP parental cells with either si-PACT#4, si-TRBP or si-NC; and PACT KO (CRISPR) cells with either si-TRBP or si-NC, and assessed TRBP expression, cell proliferation using a Cell Titer assay, colony formation, and cell cycle. When PACT was depleted from LNCaP cells, there was an increase in the expression of TRBP (Fig. 6A). Parental LNCaP cells exhibited a substantial growth reduction with si-PACT or si-TRBP as compared to the si-NC transfected cells, with the levels comparable to PACT KO cells (Fig. 6B(i)). Additionally, in the PACT KO cells, when there was also a depletion of TRBP the cells exhibited extremely poor viability and very few colonies formed in clonogenic assays (Fig. 6B(ii)). Cell cycle analysis of parental LNCaP cells transfected with each gene-specific siRNA or si-NC, or PACT KO cells transfected with si-TRBP or si-NC showed that PACT knockdown induced cell cycle arrest at G0/G1, whereas TRBP knockdown had no effect on cell cycle in parental cells, and a modest, yet significant (# *p* < 0.05) additive effect in the PACT KO cells (Fig. 6C).

Taken together, these data suggest that PACT is essential for LNCaP PCa cell growth, and TRBP expression can compensate when PACT is depleted from these cells, such that they remain viable.

## Discussion

There is a growing body of evidence that supports a requisite role for PACT in facilitating cellular growth and proliferation in tissue development and in numerous cancers (e.g., breast, liver and colorectal)^23–27,31–36^, however little is known about the role of PACT in PCa. Here we used a loss-of-function approach to characterize PACT’s mode of action in PCa and found that the depletion of PACT from PCa cell lines resulted in a reduction in cell proliferation, cell cycle arrest at G0/G1, and an increase in apoptosis. Additionally, there was a decrease in various biological processes and gene sets pertaining to cellular growth and proliferation, and to nuclear receptor (NR) function (e.g., androgen response) in the PACT depleted cells. Further, the expression of the androgen-regulated PSA gene was downregulated in the PACT knockout cells, supporting the role of PACT in modulating key androgen receptor (AR)-dependent molecules, the targeting of which could abrogate the proproliferative function of PACT in PCa.

The importance of PACT in the endocrine system has been demonstrated in mice where PACT depletion impaired the postnatal development of the anterior pituitary lobe, leading to decreased hormone levels, and defects in the ovaries, mammary glands, and in fertility^25^. NRs, such as AR, control the expression of genes and gene networks involved in cellular energy production at a transcriptional level^42^, and our TCGA PCa patient data supports this notion as the biological processes of the top-most genes which co-express with PACT are associated with energy production, such as mitochondrial and metabolic pathways. Congruent with the patient data, when PACT was knocked out of LNCaP cells, the depleted hallmark gene sets included fatty acid metabolism, reactive oxygen species pathway, and cholesterol homeostasis. Our data suggests that in addition to PACT’s widely recognized roles with PKR and the RISC complex, PACT may also have a prominent role in hormone response and facilitation of the NR-related cellular function in PCa cells, and is consistent with our previous finding that PACT is a NR co-activator in PCa cells^30^.

PSA is commonly used as a biomarker for PCa detection and disease progression, and is transcriptionally induced by androgens via AR^43,44^. There is substantial support for the clinical targeting of PSA and other kallikreins e.g., KLK2 and KLK4, as therapies in PCa^45–47^. Niu and colleagues reported that tissue PSA, independent of its protease activity, is responsible for AR-mediated PCa tumour growth^45^, and our data using either siPACT or siPSA showing reduced cell proliferation, and cell cycle arrest at G0/G1, lends further support to the concept of PSA as a therapeutic target in PCa.

The siRNA-mediated reduction of PACT decreased the proliferation of not only AR-responsive PCa cells, but also in the AR-independent PCa cells PC3 and DU145 (Supplementary Fig. S1B-C). *GATA2* and *GREB1* are coregulators of AR and have been associated with resistance to anti-androgen therapy^37^. Depletion of PACT in PC3 and DU145 cells reduced cell proliferation, while *GATA2* and *GREB1* expression levels remained unchanged. RNA-Seq analysis (Fig. 3) suggests that PACT regulates a broad range of biological processes related to cell cycle and proliferation, indicating that its function extends beyond its role as an AR coregulator.

We validated five of the most downregulated genes in LNCaP PACT KO cells (*H2AFJ, PSMD5, AQP3, TMEM45B*, and *SLC22A3*), and siRNA mediated targeting of these genes in LNCaP cells recapitulated the functional effects of PACT knockdown, and furthermore in the context of this study, these genes have reported roles as oncogenes in cancer. H2AFJ is involved in nucleosome DNA packaging into chromatin, and is implicated as an oncogene in various cancers; it is overexpressed in luminal breast and prostate cancers^48^, and its elevated levels in glioblastoma multiforme, and colorectal cancer, is associated with therapy resistance^49,50^. PSMD5 is a component of the 26S proteosome involved in cellular protein degradation, and in the context of cancer, high levels of PSMD5 expression in patients with prostate cancer had poorer overall survival^51^, and in multiple myeloma PSMD5 promoter hypermethylation resulted in resistance to proteosome inhibitors^52^. In contrast, PSMD5 was reported to be reduced in colorectal tumourigenesis and silenced with disease progression^53^. AQP3, TMEM45B, and SLC22A3 are all proteins involved in transmembrane transport of various drugs and molecules, and their aberrant overexpression has been implicated in tumourigenesis in numerous cancers (e.g., colorectal, lung, and pancreatic^54–56^; gastric, and lung^57,58^; and lung, colorectal, and prostate^59–61^, respectively). Significantly, in prostate cancer, AQP3 has been reported to promote cell motility and invasion, and its inhibition increases the sensitivity of cancer cells to cryotherapy^62,63^. Furthermore, both AQP3 and TMEM45B have potential as predictive biomarkers for PCa progression and metastasis^64,65^. Additionally, AQP3 and SLC22A3 were identified as androgen-responsive genes via RNA expression profiling of a normal prostate epithelial cell line^66^. The molecular mechanisms by which PACT regulates these genes remains unclear, with more detailed and extensive studies required to determine if this is via direct transcriptional control, miRNA processing or indirect means. Further, although we hypothesize that the changes in the expression of these target genes is a net effect of PACT loss, more research is necessitated to elucidate the benefits of targeting these genes individually in prostate cancer.

RASSF2, NOVA1, and PXDN were upregulated in the LNCaP PACT KO cells. In a variety of tumours, including prostate and lung, RASSF2 is inactivated by promoter methylation and has tumour suppressor properties^67,68^, supporting our observation of increased RASSF2 in the less proliferative PACT KO cells. In contrast, NOVA1 and PXDN are potential oncogenes in numerous cancers (e.g., head and neck carcinoma and lung cancer; and ovarian and prostate cancers, respectively) with their overexpression being associated with poor prognoses and tumour progression^69–72^.

The prostate cancer cells with either PACT depletion or knockout showed a reduction in proliferation but were viable. PACT and TRBP within the RISC complex regulate the abundance and biogenesis of microRNAs; however, each of them is dispensable in the absence of the other, and their precise role in microRNA processing is not fully understood^73,74^. PACT and TRBP interact with PKR and RIG-1 to regulate stress response, viral defense, apoptosis and gene expression, albeit in opposing manners^20,22,75,76^. Cellular stress leads to PACT phosphorylation and initiation of PKR-dependent apoptosis^25^, while overexpression and hyperphosphorylation of TRBP inhibits oxidative stress-induced apoptosis and promotes cell survival via PKR inhibition^77^. We hypothesize that the compensatory overexpression of TRBP and the subsequent inhibition of PKR may have maintained PACT-depleted PCa cell viability, and indeed the depletion of TRBP in LNCaP PACT KO cells considerably reduced cell survival. More studies are needed to comprehensively decipher the changes in the molecular landscape in the absence of PACT in PCa cells.

There is an urgent need for new treatments for advanced prostate cancer. RNA-based therapeutics is a rapidly advancing field, with the recent FDA approval of several siRNA based drugs e.g., *Inclisiran*^78^. In this context, our data provide a foundation for further work to explore siPACT or siPSA as RNA-based therapeutics for the treatment of CRPC, as their siRNA mediated targeting results in a decrease in PCa cell growth and proliferation. In addition, site-specific delivery of these siRNAs to the prostate could be achieved by harnessing PSMA through nanoparticle- or microbubble-guided ultrasound technologies, with therapeutic efficacy further enhanced by the slow, sustained release of these siRNAs from nanoparticles^15–19^, ultimately enabling more effective treatment for patients with PCa.

## Materials and methods

All the experimental protocols were performed in accordance with institutional guidelines and regulations of the Harry Perkins Institute of Medical Research and the University of Western Australia.

### Cell culture

LNCaP, C4-2B, 22Rv1, PC3, and DU145 PCa cells were from the American Type Culture Collection and cultured with RPMI-1640 (phenol free) supplemented with 10% fetal bovine serum (FBS). VCaP cells were a gift from Gail Risbridger (Monash University, Melbourne, Australia), and were grown in DMEM (high glucose) + 10% FBS. All cells were cultured at 37˚C in 5% CO_2_.

### Transfection of siRNA molecules

Cells were seeded into 6-well or 10 cm dishes and transfected with 20 nM siRNA using Lipofectamine 2000 (Thermo Fisher Scientific) according to the manufacturer’s instructions. Silencer Select siRNAs to *PRKRA* (IDs: s16334 and s16336), *H2AFJ* (IDs: s31461, s31462 and s31463), *AQP3* (IDs: s1521, s1522 and s1523), *TMEM45B* (IDs: s42358, s42359 and s42360), *SLC22A3* (IDs: s13105, s13106 and s13107), *TRBP2* (IDs: s13790, s13791 and s13792), and a negative control siRNA (Cat #4390843) were from Thermo Fisher Scientific. siRNA FlexiTube GeneSolution for *PSMD5* (GS5711) and *KLK3* (GS354) were from Qiagen.

### RNA extraction, reverse transcription and quantitative polymerase chain reaction (RT-qPCR)

Total RNA was extracted from cells using Trizol reagent (Thermo Fisher Scientific) as per the manufacturer’s instuctions. Total RNA (800 ng) was used to generate cDNA using a QuantiTect Reverse Transcription Kit (Qiagen), and PCR performed using SYBR SensiMix (Bioline) and the following QuantiTect primer assays (Qiagen): Hs_PRKRA_1_SG, Hs_GATA2_1_SG, Hs_GREB1_1_SG, Hs_H2AFJ_1_SG, Hs_PSMD5_1_SG, Hs_AQP3_1_SG, Hs_TMEM45B_1_SG, Hs_SLC22A31_1_SG, Hs_NOVA1_1_SG, Hs_PXDN_1_SG, Hs_RASSF2_1_SG, Hs_KLK3_1_SG, Hs_KLK2_1_SG, Hs_KLK4_1_SG, Hs_TARBP2_1_SG, Hs_GAPDH_2_SG, and Hs_HPRT1_1_SG. The 2^−ΔΔCt^ method was used to determine normalised gene expression.

### Protein extraction and western immunoblotting

Whole cell protein lysates were prepared using mid-RIPA lysis buffer (50 mM Tris (pH 7.4), 150 mM NaCl, 1% NP-40, 1% sodium deoxycholate, 0.1% SDS) and western blotting performed. Briefly, lysates were resolved on NuPAGE 4–12% Bis–Tris gels (Thermo Fisher Scientific) and transferred to PVDF membranes. Membranes were blocked in 5% skim milk/Tris-buffered saline Tween 20 and probed with antibodies to PACT (Santa Cruz sc-18768), PSA (DAKO A0562), β-actin (AbCam ab6276), or tubulin (AbCam ab4074). Protein detection was with horseradish peroxidase-linked anti-goat IgG (AbCam ab6885), anti-mouse IgG (Amersham NA931), or anti-rabbit IgG (Amersham NA934) with Luminata Classico Western HRP substrate (Millipore), and visualization was with either ECL-Hyperfilm (GE Healthcare) or the iBright Imaging System (Thermo Fisher Scientific).

### Targeted knockout of PACT using CRISPR-Cas 9

LNCaP and C4-2B PCa cells were grown to ~ 80% confluency in 10 cm dishes and transfected with 5 ug PACT CRISPR all in one vector gRNA + Cas9WT plasmid (Sigma; CRISPR target ID: HS0000133877; target gene ID: 8575 (*PRKRA*)) using Lipofectamine 2000. 72 h post-transfection green fluorescent protein (GFP) expressing cells were isolated by FACS (FACSCAlibur, BD Biosciences) and seeded at low density in 10 cm dishes. Cells were grown until single colonies formed and selected clones verified to contain the PACT knockdown via western blotting, PCR, and DNA sequencing (see Supplementary Fig. S1F for primers).

### Stable overexpression and reconstitution of PACT in parental and PACT knockout LNCaP cells

LNCaP parental and PACT KO cells stably expressing PACT cDNA (pcDNA-PACT; a gift from Dong-Yan Jin (Addgene plasmid #15667; http://n2t.net/addgene:15667; RRID:Addgene_15667)^79^ were generated by lentiviral transduction as previously described^80^. Briefly, cells were infected with lentiviruses carrying LeGO-iG2-Empty (a gift from Boris Fehse (Addgene plasmid #27341; http://n2t.net/addgene:27341; RRID:Addgene_27341)^81^ or LeGO-iG2-pcDNA-PACT plasmids, and transduced cells stably expressing GFP were isolated by FACS. Validation of the ectopic expression of PACT in the isolated cells was with western blotting.

### Cell proliferation and colony forming assays

For cell growth and proliferation assays PCa cells (± 24 h siRNA transfection, as described above) were plated at a density of 5000 cells/well in either; 96 well plates and growth assessed at end-point 1–7 days post-seeding using a CellTiter 96 AQ_ueous_ One Solution Cell Proliferation Assay (Promega) and the Fluostar OPTIMA microplate reader (BMG Scientific), or into 16 well xCELLigence E-plates and measurement of cell proliferation in real time using the xCELLigence instrument (ELITechGroup). For colony forming assays, cells were plated at a low density (1 × 10^4^ cells in a 10 cm dish) and colonies were allowed to develop for ~ 2 weeks prior to staining with crystal violet for visualization as previously described^82^.

### Hormone treatment

2 × 10^5^ LNCaP or C4-2B parental, PACT KO, or PACT overexpressing cells were plated into 6-well plates and allowed to settle overnight. Cells were replenished in media supplemented with 10% charcoal stripped FBS for 24 h prior to treatment with 10 nM DHT (dihydrotestosterone, Cayman Chemicals), 10 nM R1881 (metribolone, Sigma), 1 uM MDV3100 (enzalutamide, MedChemExpress), or DMSO (Sigma) vehicle control. Harvesting of RNA or protein was at 24 h post-treatment.

### RNA-sequencing (RNA-seq)

For RNA-seq analysis, 1 × 10^6^ LNCaP parental and LNCaP PACT CRISPR KO cells were plated into 10 cm dishes (in triplicate) and when the cells were ~ 80% confluent total RNA was extracted using Trizol. Confirmation of the quantity and integrity of extracted RNA was with the 2100 Bioanalyzer (Agilent Technologies), and gene expression profiling was performed at the Australian Genome Research Facility (AGRF) using the Illumina NovaSeq platform and standard protocols. The differential gene expression analysis was performed using edgeR (v3.22.3) and default TMM normalization methods.

### Clinical datasets and pathway analyses

The publically available Prostate Adenocarcinoma datasets contained within The Cancer Genome Atlas (TCGA), PanCancer Atlas (Cell, 2018), n = 494 patients, were accessed via the cBioPortal for Cancer Genomics (https://www.cbioportal.org/). Database for Annotation, Visualization and Integrated Discovery (DAVID; version 2021) was used to perform gene ontology (GO) biological processes and KEGG (Kyoto Encyclopedia of Genes and Genomes)^40^ pathway analyses. Gene Set Enrichment Analysis (GSEA), incorporating the MSigDB (Human Molecular Signatures Database), was used to perform GO biological processes and Hallmark gene set analyses of the RNA-Seq data, as previously described^83,84^.

### Cell cycle analysis and annexin V-APC/PI apoptosis assay

LNCaP PCa cells were transfected as described above with 20 nM gene specific siRNA or si-NC for 72 h, followed by collection of both floating and adherent cells for assaying. For cell cycle analysis cells were fixed with cold 100% ethanol, and stained with Propidium Iodide (PI) staining solution (25 µg/ml PI and 0.25 µg/ml RNase A in PBS). For assessment of apoptosis, the Annexin V-APC/PI Apoptosis Detection Kit I (BD Biosciences) was used according to manufacturer’s instructions, with no stain, single stain and Camptothecin (10 µM, 24 h (Cayman Chemicals)) treated cells used to set gating strategies. Samples for both assays were analysed using the BD Accuri C6 Flow Cytometer and FlowJo Software (version 7.6.5). For LNCaP parental versus PACT KO cells, cells were seeded into 10 cm dishes and assayed as above when ~ 80% confluent.

### Statistical analysis

Graphing and analysis of data was with GraphPad Prism v.10 software. Use of the unpaired *t*-test (two-tailed) determined significant differences in RT-qPCR, cell proliferation, colony formation, apoptosis, cell cycle analyses. Significance levels are indicated as: *p < 0.05, or **p < 0.005.

## Supplementary Information

Below is the link to the electronic supplementary material.

Supplementary Material 1

Supplementary Material 2

Supplementary Material 3

Supplementary Material 4

Supplementary Material 5

Supplementary Material 6

## Data Availability

The RNA-Seq data is available in the Gene Expression Omnibus under Accession Number GSE253245 (https://www.ncbi.nlm.nih.gov/geo/query/acc.cgi?acc=GSE253245). Enter token slgnmoacptktzcr into the box.

## Abbreviations

PACT: Protein kinase RNA activator
PCa: Prostate cancer
RNA-Seq: RNA-sequencing
NR: Nuclear receptor
siRNA: Small interfering RNA
RT-qPCR: Reverse transcription and quantitative polymerase chain reaction
KO: Knockout
PSA: Prostate specific antigen
AR: Androgen receptor
CRPC: Castrate resistant prostate cancer
PKR: Protein kinase RNA
TRBP: Transactivation response RNA binding protein
OE: Overexpression
DHT: Dihydrotestosterone
PSMA: Prostate specific membrane antigen
