# Supplementary material for: PACT is requisite for prostate cancer cell proliferation
Source: Sci Rep. 2025 Oct 21;15:36610. doi: 10.1038/s41598-025-20494-9 (PMC12540807; doi:10.1038/s41598-025-20494-9)
Supplement: Supplementary file 1 — Supplementary Material 1 [file 41598_2025_20494_MOESM1_ESM.docx]

# PACT is requisite for Prostate Cancer cell proliferation

**Supplementary Tables and Figure Legends**

**Supplementary Table 1.** A list of the top 200 genes that positively correlate to *PRKRA*/PACT mRNA expression in the Prostate Adenocarcinoma TCGA PanCancer Atlas (p<0.05, ranked highest to lowest using Spearman’s correlation).

**Supplementary Table 2.** A list of the top 200 genes that negatively correlate to *PRKRA*/PACT mRNA expression in the Prostate Adenocarcinoma TCGA PanCancer Atlas (p<0.05, ranked highest to lowest using Spearman’s correlation).

**Supplementary Table 3.** Downregulated genes in the PACT KO cells as compared to parental LNCaP. The genes validated and further investigated herein are in bold and are namely: *H2AFJ* (H2A histone family member J)*, PSMD5* (proteasome 26s non-ATPase subunit, 5)*, AQP3* (Aquaporin 3), *TMEM45B* (transmembrane protein 45B), *SLC22A3* (Solute carrier family 22 member 3), *KLK3* (Kallikrein related peptidase 3, Prostate specific antigen, PSA), and *KLK2* (Human kallikrein), and were respectively downregulated 10.76, 7.18, 5.88, 4.63, 3.9, 2.49, 0.83 log_2_ fold change in the PACT KO cells.

**Supplementary Table 4.** Upregulated genes in the PACT KO cells as compared to parental LNCaP. The genes validated and further investigated herein are in bold and are namely: *NOVA1* (neuro-oncological ventral antigen-1), *PXDN* (Peroxidasin), and *RASSF2* (Ras association domain-containing protein 2), and were respectively upregulated 2.77, 2.33, and 1.4 log_2_ fold change, in the PACT KO cells.

**Supplementary Figure S1. PACT sustains proliferation in prostate cancer cells; and PCR and sequencing of CRISPR PACT KO clones in LNCaP cells. (A)** 22Rv1 **(B)** PC3 **(C)** DU145 or **(D)** VCaP PCa cells were treated with negative control siRNA (si-NC) or PACT siRNA (si-PACT#4) for one day and then harvested for RNA extraction or for seeding into appropriate plates for 72 h for **(ii)** cell titre proliferation assay; or **(iii)** xCELLigence assay. **(i)** RT-qPCR validation of si-PACT knockdown at 1 d post-transfection. **(E)** PC3 **(i)** or DU145 **(ii)** cells were transfected with si-NC or si-PACT#4 for 24 h and harvested for RNA extraction. RT-qPCR was used to validate si-PACT knockdown and its effect on GREB1 and GATA2 mRNA expression. Expression of gene specific mRNA is normalised to GAPDH or HPRT housekeeping gene expression, calculated using the 2^-ΔΔCt^ method, and relative to si-NC. Error bars = SE (RT-qPCR) or SD (cell proliferation); n = 3; data analyses used an unpaired two-tailed student’s t-test; with significance denoted as: *p<0.05, **p<0.005 relative to si-NC. **(F) (i)** Schematic of the PCR and sequencing primers, and the location and sequence of the CRISPR site within Exon 2 of the *PACT* (*PRKRA*) gene; **(ii)** PCR identification of clones 32 and 21 as homozygous knockouts of *PACT*, as the bottom band failed to amplify with primers PACT#2F and PACT#3R; and **(iii)** Sequencing of Clone 21 using PACT#1F primers to verify the CRISPR mediated deletion of PACT. Clone 21 was used in all subsequent experiments**.**

**Supplementary Figure S2. PACT expression positively correlates with genes involved in metabolic processes. (A) (i)** Gene ontology (GO) Biological Processes analyses of the top 200 genes whose expression was positively correlated with PACT expression (P<0.05, and Spearman’s correlation values highest to lowest), and **(ii)** ATP5BP gene expression positively correlated to PACT mRNA expression. **(B) (i)** GO Biological Processes analyses of the top 200 genes whose expression was negatively correlated with PACT expression (P<0.05, and Spearman’s correlation values highest to lowest), and **(ii)** Expression of ASLX1 negatively correlated to PACT expression. The mRNA expression was batch normalized from Illumina HISeq_RNA Seq V2, and the Spearman’s correlation, p-values, and q-values are indicated in Supplementary Tables 1 and 2.

**Supplementary Figure S3. RT-qPCR validation of siRNA mediated knockdown of gene expression.** RT-qPCR analysis of normalized gene expression calculated using the 2^-ΔΔCt^ method, and relative to si-NC. Housekeeping genes were HPRT or GAPDH. LNCaP cells were transfected with 20 nM gene specific siRNA or a negative control siRNA (si-NC) and RNA extraction was at 24 h post transfection. **(A)** Initial testing of three or four different gene specific siRNAs for **(i)** H2AFJ; **(ii)** PSMD5; **(iii)** AQP3; **(iv)** TMEM45B; and **(v)** SLC22A3. The boxes around the specific siRNAs indicate the siRNAs that were used in subsequent experiments e.g., blue box around H2AFJ s31462. **(B)** Validation of gene specific siRNA mediated knockdown for Cell Titer assay reported in Fig. 2C. **(C)** Validation of si-PACT knockdown for cell cycle and apoptosis assays reported in Fig. 3D (i) and Fig. 3E (i), respectively. Error bars = SE; Unpaired two-tailed *t*-test; significance denoted as: *p<0.05, **p<0.005 relative to si-NC.

**Supplementary Figure S4. Western blot validation of PACT knockout/overexpression and PSA expression; RT-qPCR validation of siRNA mediated knockdown of PSA gene expression; functional assays of si-PSA in 22Rv1 PCa cells; and PSA expression in a panel of PCa cell lines. (A)** LNCaP parental and PACT KO cells were treated with 10 nM DHT for 24 h and PSA protein expression measured using western blotting. **(B)** C4-2B parental and PACT KO cells were treated with 10 nM R1881 for 24 h. **(i)** western blot of PACT expression verifying knockdown, and **(ii)** western blot of PSA protein expression. **(C)** Western blot of LNCaP cells overexpressing empty vector (EV) or PACT (OE). For all westerns β-actin is the loading control and see Supplementary Fig. S6D and F-H for original non-cropped westerns. **(D)** RT-qPCR analysis of LNCaP cells transfected with 20 nM of four different PSA siRNA constructs or a negative control siRNA (si-NC). The green box denotes the selection of si-PSA SI03078299 for use in subsequent experiments. **(E-F)** LNCaP or C4-2B cells were transfected with 20 nM si-PSA SI03078299 or si-NC for functional assays reported in Fig. 5D-E **(i)** cell proliferation; **(ii)** colony formation; and **(iii)** cell cycle analyses. **(G)** 22Rv1 cells were transiently transfected with 20 nM PSA siRNA (si-PSA) or negative control siRNA (si-NC) and assayed for the effects of gene knockdown via **(i)** cell proliferation at 3 d and 6 d post-transfection; **(ii)** colony formation at ~ 2 weeks post-transfection; and **(iii)** cell cycle analyses. **(iv)** RT-qPCR validation of PSA gene knockdown 72 h post-transfection. **(H)** RT-qPCR comparison of basal PSA expression in prostate cancer cell lines. Expression of PSA mRNA is normalised to GAPDH or HPRT housekeeping gene expression, calculated using the 2^-ΔΔCt^ method, and relative to si-NC or LNCaP cells. Error bars = SE (RT-qPCR) or SD (G (i-iii)); n = 3; unpaired two-tailed *t*-test; significance denoted as: *p<0.05, **p<0.005 relative to si-NC or LNCaP cells.

**Supplementary Figure S5. RT-qPCR validation of siRNA mediated knockdown of TRBP or PACT gene expression (A)** LNCaP cells were transfected with 20 nM of three different TRBP siRNA constructs or a negative control siRNA (si-NC). The blue box denotes the selection of TRBP s13790 siRNA for use in subsequent experiments. **(B)** LNCaP parental and LNCaP PACT CRISPR KO cells were transfected with 20 nM si-TRBP s13790, or si-PACT s16344 (LNCaP parental cells), or si-NC for functional assays reported in Fig. 6; **(i)** TRBP gene expression and cell proliferation; **(ii)** colony formation; and **(iii)** cell cycle analyses. Expression of TRBP mRNA is normalised to GAPDH housekeeping gene expression, calculated using the 2^-ΔΔCt^ method, and relative to si-NC parental LNCaP. Error bars = SE; n = 3; unpaired two-tailed *t*-test; significance denoted as: *p<0.05, **p<0.005 relative to si-NC parental cells

**Supplementary Figure S6. Original western blots of PACT KO, overexpression, and PSA expression ± DHT/R1881 treatment. (A)** LNCaP or **(B)** C4-2B PCa cells were treated with negative control siRNA (si-NC) or two different PACT siRNAs (si-PACT#4 and si-PACT#6) for 72 h and protein extracted for western blot analysis of PACT expression, β-actin is the loading control. The blots shown here are the original images used to generate Figure 1A (i) and Figure 1B (i), with the red boxes indicating the lanes used. **(C-D)** Western blot analysis of PACT expression in parental LNCaP or C4-2B cells and LNCaP or C4-2B PACT CRISPR knockout (KO), tubulin is the loading control. The blots shown here are the original non-cropped blots used to generate Fig. 1C (i) and Supplementary Fig. S4B (i), with the red boxes indicating the lanes used. **(E -F)** Western blot analysis of LNCaP parental and CRISPR PACT KO cells stably overexpressing empty vector (EV) or PACT cDNA (PACT OE), β-actin loading control. The blots shown here are the original non-cropped blots used to generate Fig. 1D (i) and Supplementary Fig. S4C, with the red boxes indicating the lanes used. **(G)** LNCaP or **(H)** C4-2B parental or PACT KO cells were plated into 6 well plates and allowed to settle overnight. Cells were grown for 6-24 h in media supplemented with 10% charcoal stripped FBS prior to treatment with 10 nM DHT/R1881 or DMSO vehicle control, protein was harvested from the cells 24 h post treatment and subjected to western blot analysis for PSA protein expression, β-actin loading control. The blots shown here are the original non-cropped blots used to generate Supplementary Fig. S4A and B (ii), with the red boxes indicating the lanes used.
