# Supplementary material for: PACT is requisite for prostate cancer cell proliferation
Source: Sci Rep. 2025 Oct 21;15:36610. doi: 10.1038/s41598-025-20494-9 (PMC12540807; doi:10.1038/s41598-025-20494-9)
Supplement: Supplementary file 3 — Supplementary Material 3 [file 41598_2025_20494_MOESM3_ESM.docx]

**Supplementary Table 1.** A list of the top 200 genes that positively correlate to *PRKRA*/PACT mRNA expression in the Prostate Adenocarcinoma TCGA PanCancer Atlas (p<0.05, ranked highest to lowest using Spearman’s correlation).

| Correlated Gene | Spearman's Correlation | p-Value | q-Value |
| --- | --- | --- | --- |
| *PLEKHA3* | 0.460021 | 3.46E-27 | 6.92E-23 |
| *PSMD14* | 0.420126 | 1.67E-22 | 1.66E-18 |
| *SSB* | 0.418538 | 2.50E-22 | 1.66E-18 |
| *ATP5MC3* | 0.404249 | 8.34E-21 | 4.17E-17 |
| *BCAS2* | 0.391328 | 1.73E-19 | 5.75E-16 |
| *HNRNPA3* | 0.387982 | 3.70E-19 | 1.06E-15 |
| *SUMO1* | 0.380464 | 1.99E-18 | 4.98E-15 |
| *MTX2* | 0.373752 | 8.63E-18 | 1.92E-14 |
| *COQ10B* | 0.370414 | 1.77E-17 | 3.53E-14 |
| *RAB1A* | 0.367298 | 3.42E-17 | 6.22E-14 |
| *SUB1* | 0.366122 | 4.39E-17 | 7.31E-14 |
| *MRPS36* | 0.364158 | 6.62E-17 | 1.02E-13 |
| *ESD* | 0.362654 | 9.05E-17 | 1.29E-13 |
| *UCHL3* | 0.359625 | 1.69E-16 | 2.26E-13 |
| *MICU2* | 0.353984 | 5.33E-16 | 6.28E-13 |
| *TMEM126B* | 0.346952 | 2.16E-15 | 2.40E-12 |
| *HNRNPA3P1* | 0.342727 | 4.93E-15 | 4.69E-12 |
| *ATP5PB* | 0.342397 | 5.25E-15 | 4.77E-12 |
| *TPMT* | 0.341551 | 6.18E-15 | 5.15E-12 |
| *SUMO1P3* | 0.340021 | 8.29E-15 | 6.63E-12 |
| *SUCLG1* | 0.339481 | 9.20E-15 | 7.07E-12 |
| *ATP5F1C* | 0.336942 | 1.49E-14 | 1.06E-11 |
| *CALM2* | 0.333615 | 2.79E-14 | 1.84E-11 |
| *COPS8* | 0.333324 | 2.95E-14 | 1.84E-11 |
| *NDUFB3* | 0.331845 | 3.88E-14 | 2.30E-11 |
| *UBE2A* | 0.330341 | 5.13E-14 | 2.93E-11 |
| *SAP18* | 0.329491 | 6.01E-14 | 3.34E-11 |
| *MOCS2* | 0.327831 | 8.15E-14 | 4.38E-11 |
| *METTL5* | 0.327578 | 8.53E-14 | 4.38E-11 |
| *VBP1* | 0.326859 | 9.73E-14 | 4.87E-11 |
| *SELENOF* | 0.324587 | 1.47E-13 | 7.18E-11 |
| *SNRNP27* | 0.324124 | 1.60E-13 | 7.62E-11 |
| *MRPS10* | 0.323748 | 1.71E-13 | 7.96E-11 |
| *VPS45* | 0.322943 | 1.98E-13 | 9.00E-11 |
| *ATP6V1D* | 0.321183 | 2.71E-13 | 1.18E-10 |
| *VAPA* | 0.320974 | 2.82E-13 | 1.20E-10 |
| *GORASP2* | 0.319948 | 3.38E-13 | 1.41E-10 |
| *GTF2A2* | 0.319407 | 3.73E-13 | 1.52E-10 |
| *FDX1* | 0.318675 | 4.24E-13 | 1.70E-10 |
| *EIF4E* | 0.316139 | 6.64E-13 | 2.60E-10 |
| *ZBTB8OS* | 0.315872 | 6.95E-13 | 2.67E-10 |
| *SELENOT* | 0.315421 | 7.53E-13 | 2.74E-10 |
| *ERH* | 0.315206 | 7.81E-13 | 2.79E-10 |
| *PTS* | 0.314629 | 8.64E-13 | 3.03E-10 |
| *MORF4L2* | 0.311526 | 1.48E-12 | 5.11E-10 |
| *MDH1* | 0.311336 | 1.53E-12 | 5.15E-10 |
| *DYNLT1* | 0.311276 | 1.55E-12 | 5.15E-10 |
| *MTCH2* | 0.31092 | 1.64E-12 | 5.39E-10 |
| *PSMC6* | 0.310074 | 1.90E-12 | 6.04E-10 |
| *ACTR10* | 0.308742 | 2.39E-12 | 7.23E-10 |
| *POMP* | 0.307531 | 2.93E-12 | 8.76E-10 |
| *HIBCH* | 0.306405 | 3.55E-12 | 1.04E-09 |
| *RTN4* | 0.304193 | 5.15E-12 | 1.49E-09 |
| *HAT1* | 0.303646 | 5.65E-12 | 1.61E-09 |
| *DNAJC8* | 0.301586 | 7.96E-12 | 2.15E-09 |
| *SELENOK* | 0.299882 | 1.06E-11 | 2.78E-09 |
| *RAP1A* | 0.299135 | 1.19E-11 | 3.10E-09 |
| *UTP11* | 0.298894 | 1.24E-11 | 3.16E-09 |
| *TWF1* | 0.298874 | 1.25E-11 | 3.16E-09 |
| *MCUR1* | 0.297088 | 1.67E-11 | 4.18E-09 |
| *AIG1* | 0.295548 | 2.15E-11 | 5.30E-09 |
| *SRP9* | 0.294864 | 2.40E-11 | 5.78E-09 |
| *VPS29* | 0.292973 | 3.25E-11 | 7.42E-09 |
| *SEC22B* | 0.292948 | 3.27E-11 | 7.42E-09 |
| *NUDT15* | 0.292031 | 3.78E-11 | 8.22E-09 |
| *PDZD11* | 0.29145 | 4.15E-11 | 8.93E-09 |
| *OCIAD1* | 0.291091 | 4.40E-11 | 9.34E-09 |
| *ZC3H15* | 0.291032 | 4.44E-11 | 9.34E-09 |
| *GRIK1* | 0.289541 | 5.62E-11 | 1.15E-08 |
| *TXNDC9* | 0.287735 | 7.48E-11 | 1.47E-08 |
| *NDUFA5* | 0.287194 | 8.14E-11 | 1.58E-08 |
| *COA5* | 0.286043 | 9.75E-11 | 1.88E-08 |
| *AAGAB* | 0.285855 | 1.00E-10 | 1.91E-08 |
| *SCFD1* | 0.285774 | 1.02E-10 | 1.92E-08 |
| *MPV17* | 0.285317 | 1.09E-10 | 2.02E-08 |
| *LIPT1* | 0.285043 | 1.14E-10 | 2.09E-08 |
| *SCOC* | 0.284794 | 1.18E-10 | 2.15E-08 |
| *SPTSSA* | 0.284762 | 1.19E-10 | 2.15E-08 |
| *SRP14* | 0.284401 | 1.26E-10 | 2.25E-08 |
| *ZFAND6* | 0.284057 | 1.33E-10 | 2.34E-08 |
| *TANK* | 0.283461 | 1.46E-10 | 2.53E-08 |
| *MOB4* | 0.281268 | 2.04E-10 | 3.43E-08 |
| *PCBP1* | 0.280363 | 2.35E-10 | 3.88E-08 |
| *TMEM165* | 0.280214 | 2.40E-10 | 3.93E-08 |
| *POLR2K* | 0.279772 | 2.57E-10 | 4.14E-08 |
| *FAM174A* | 0.279495 | 2.68E-10 | 4.29E-08 |
| *MED4* | 0.279363 | 2.73E-10 | 4.34E-08 |
| *ARL1* | 0.279077 | 2.85E-10 | 4.47E-08 |
| *ARG2* | 0.279062 | 2.86E-10 | 4.47E-08 |
| *TMEM50A* | 0.278595 | 3.07E-10 | 4.76E-08 |
| *PIFO* | 0.278486 | 3.12E-10 | 4.80E-08 |
| *SDHB* | 0.278202 | 3.26E-10 | 4.98E-08 |
| *EID2* | 0.278133 | 3.29E-10 | 4.99E-08 |
| *PPP2CA* | 0.277747 | 3.49E-10 | 5.21E-08 |
| *GTF2F2* | 0.277534 | 3.61E-10 | 5.34E-08 |
| *OLA1* | 0.277454 | 3.65E-10 | 5.37E-08 |
| *CAMKMT* | 0.2773 | 3.74E-10 | 5.45E-08 |
| *WDR61* | 0.27719 | 3.80E-10 | 5.50E-08 |
| *SERP1* | 0.27704 | 3.88E-10 | 5.59E-08 |
| *TMEM59* | 0.276815 | 4.02E-10 | 5.74E-08 |
| *RBM45* | 0.276394 | 4.28E-10 | 6.06E-08 |
| *PPCS* | 0.276214 | 4.40E-10 | 6.11E-08 |
| *MORF4L1* | 0.275014 | 5.27E-10 | 7.16E-08 |
| *RIDA* | 0.274474 | 5.71E-10 | 7.71E-08 |
| *SDHD* | 0.274408 | 5.76E-10 | 7.74E-08 |
| *CRIPT* | 0.27433 | 5.83E-10 | 7.78E-08 |
| *APOO* | 0.274048 | 6.08E-10 | 8.00E-08 |
| *UPRT* | 0.273995 | 6.13E-10 | 8.01E-08 |
| *SMIM15* | 0.273863 | 6.25E-10 | 8.12E-08 |
| *TIMM17A* | 0.273716 | 6.39E-10 | 8.25E-08 |
| *NAP1L5* | 0.273344 | 6.75E-10 | 8.66E-08 |
| *PIGF* | 0.272981 | 7.13E-10 | 9.02E-08 |
| *LAMTOR3* | 0.272334 | 7.85E-10 | 9.87E-08 |
| *TLK1* | 0.271437 | 8.96E-10 | 1.11E-07 |
| *TMEM230* | 0.271204 | 9.27E-10 | 1.14E-07 |
| *NUCB2* | 0.270413 | 1.04E-09 | 1.27E-07 |
| *NGRN* | 0.270136 | 1.08E-09 | 1.31E-07 |
| *SRP19* | 0.269656 | 1.16E-09 | 1.39E-07 |
| *RAB18* | 0.269572 | 1.18E-09 | 1.40E-07 |
| *EAPP* | 0.269495 | 1.19E-09 | 1.41E-07 |
| *CAMTA1* | 0.269171 | 1.25E-09 | 1.46E-07 |
| *COX7B* | 0.268525 | 1.37E-09 | 1.60E-07 |
| *C11ORF58* | 0.267461 | 1.60E-09 | 1.81E-07 |
| *SPRYD7* | 0.266128 | 1.94E-09 | 2.13E-07 |
| *COX7A2* | 0.265914 | 2.00E-09 | 2.19E-07 |
| *PRR13* | 0.265717 | 2.06E-09 | 2.24E-07 |
| *TBCA* | 0.265155 | 2.23E-09 | 2.40E-07 |
| *CCNC* | 0.264914 | 2.31E-09 | 2.47E-07 |
| *CISD2* | 0.264775 | 2.36E-09 | 2.50E-07 |
| *MPC1* | 0.264135 | 2.59E-09 | 2.67E-07 |
| *MRPL18* | 0.263856 | 2.69E-09 | 2.76E-07 |
| *GNRHR2* | 0.263328 | 2.90E-09 | 2.95E-07 |
| *DHRS13* | 0.263284 | 2.92E-09 | 2.95E-07 |
| *LACTB* | 0.262574 | 3.23E-09 | 3.25E-07 |
| *NDUFA12* | 0.262256 | 3.38E-09 | 3.36E-07 |
| *TM2D3* | 0.262035 | 3.49E-09 | 3.44E-07 |
| *PSMA4* | 0.261986 | 3.51E-09 | 3.44E-07 |
| *FAM98A* | 0.261419 | 3.80E-09 | 3.66E-07 |
| *ORC4* | 0.261339 | 3.85E-09 | 3.67E-07 |
| *C1D* | 0.261323 | 3.86E-09 | 3.67E-07 |
| *RHOQ* | 0.261259 | 3.89E-09 | 3.69E-07 |
| *PSMC4* | 0.261181 | 3.93E-09 | 3.70E-07 |
| *TTC8* | 0.261092 | 3.98E-09 | 3.72E-07 |
| *PDHB* | 0.260518 | 4.32E-09 | 3.98E-07 |
| *TMCO1* | 0.260052 | 4.61E-09 | 4.23E-07 |
| *CEBPG* | 0.259057 | 5.30E-09 | 4.78E-07 |
| *UBE2D3* | 0.258664 | 5.60E-09 | 5.03E-07 |
| *CYCS* | 0.258559 | 5.69E-09 | 5.08E-07 |
| *UBE2F* | 0.25852 | 5.72E-09 | 5.08E-07 |
| *PARK7* | 0.258476 | 5.75E-09 | 5.08E-07 |
| *WASHC3* | 0.258434 | 5.79E-09 | 5.08E-07 |
| *SCRN3* | 0.258325 | 5.88E-09 | 5.13E-07 |
| *NDUFB6* | 0.257192 | 6.88E-09 | 5.91E-07 |
| *LAMTOR5* | 0.256636 | 7.43E-09 | 6.35E-07 |
| *NDUFAF4* | 0.256474 | 7.60E-09 | 6.47E-07 |
| *STAMBP* | 0.256432 | 7.64E-09 | 6.48E-07 |
| *SCP2* | 0.256356 | 7.72E-09 | 6.52E-07 |
| *RWDD2B* | 0.256169 | 7.93E-09 | 6.66E-07 |
| *DYNLT3* | 0.256033 | 8.08E-09 | 6.76E-07 |
| *EMC7* | 0.255898 | 8.23E-09 | 6.83E-07 |
| *DERL2* | 0.255358 | 8.87E-09 | 7.32E-07 |
| *DNAJC1* | 0.25534 | 8.89E-09 | 7.32E-07 |
| *PBDC1* | 0.254961 | 9.36E-09 | 7.64E-07 |
| *SPCS2* | 0.254748 | 9.64E-09 | 7.81E-07 |
| *TP53RK* | 0.253647 | 1.12E-08 | 8.87E-07 |
| *OST4* | 0.253396 | 1.16E-08 | 9.08E-07 |
| *PDCL3* | 0.253328 | 1.17E-08 | 9.12E-07 |
| *DUSP14* | 0.253001 | 1.22E-08 | 9.50E-07 |
| *AK2* | 0.252897 | 1.24E-08 | 9.59E-07 |
| *SPAG7* | 0.252781 | 1.26E-08 | 9.71E-07 |
| *ACYP2* | 0.252632 | 1.29E-08 | 9.87E-07 |
| *CDK2AP1* | 0.252193 | 1.37E-08 | 1.04E-06 |
| *FUCA1* | 0.252181 | 1.37E-08 | 1.04E-06 |
| *GNL2* | 0.252174 | 1.37E-08 | 1.04E-06 |
| *UFSP2* | 0.252029 | 1.40E-08 | 1.06E-06 |
| *MICOS10* | 0.251554 | 1.49E-08 | 1.12E-06 |
| *PSMA3* | 0.250527 | 1.71E-08 | 1.27E-06 |
| *C14ORF119* | 0.250525 | 1.71E-08 | 1.27E-06 |
| *SNRPGP15* | 0.250333 | 1.76E-08 | 1.29E-06 |
| *UBE2N* | 0.250325 | 1.76E-08 | 1.29E-06 |
| *PNRC2* | 0.250128 | 1.81E-08 | 1.32E-06 |
| *ARF4* | 0.249759 | 1.90E-08 | 1.38E-06 |
| *FKBP1A* | 0.249712 | 1.91E-08 | 1.39E-06 |
| *RPF1* | 0.249683 | 1.92E-08 | 1.39E-06 |
| *SPCS1* | 0.249605 | 1.94E-08 | 1.4E-06 |
| *LSM3* | 0.249435 | 1.99E-08 | 1.42E-06 |
| *SNRPG* | 0.249361 | 2.01E-08 | 1.43E-06 |
| *METTL8* | 0.249077 | 2.08E-08 | 1.47E-06 |
| *BZW1* | 0.248168 | 2.35E-08 | 1.65E-06 |
| *CDC42* | 0.247267 | 2.65E-08 | 1.84E-06 |
| *ITM2B* | 0.246937 | 2.77E-08 | 1.91E-06 |
| *HCCS* | 0.246826 | 2.81E-08 | 1.93E-06 |
| *FUNDC2* | 0.246661 | 2.88E-08 | 1.96E-06 |
| *MTRES1* | 0.246305 | 3.02E-08 | 2.04E-06 |
| *TMEM167B* | 0.246244 | 3.04E-08 | 2.05E-06 |
| *COMMD1* | 0.246143 | 3.08E-08 | 2.06E-06 |
| *PPA2* | 0.246012 | 3.13E-08 | 2.08E-06 |
| *PPP1R2* | 0.245851 | 3.20E-08 | 2.12E-06 |
| *HIGD1A* | 0.245548 | 3.33E-08 | 2.19E-06 |
| *CCDC115* | 0.245404 | 3.40E-08 | 2.23E-06 |
