# Supplementary material for: PACT is requisite for prostate cancer cell proliferation
Source: Sci Rep. 2025 Oct 21;15:36610. doi: 10.1038/s41598-025-20494-9 (PMC12540807; doi:10.1038/s41598-025-20494-9)
Supplement: Supplementary file 4 — Supplementary Material 4 [file 41598_2025_20494_MOESM4_ESM.docx]

**Supplementary Table 2.** A list of the top 200 genes that negatively correlate to *PRKRA*/PACT mRNA expression in the Prostate Adenocarcinoma TCGA PanCancer Atlas (p<0.05, ranked highest to lowest using Spearman’s correlation).

| Correlated Gene | Spearman's Correlation | p-Value | q-Value |
| --- | --- | --- | --- |
| *ABCC1* | -0.39466 | 8.01E-20 | 3.20E-16 |
| *HCFC1* | -0.35876 | 2.02E-16 | 2.53E-13 |
| *TNS3* | -0.34537 | 2.94E-15 | 3.10E-12 |
| *ASXL1* | -0.34494 | 3.21E-15 | 3.21E-12 |
| *TSC22D4* | -0.34194 | 5.74E-15 | 4.99E-12 |
| *CTDSP2* | -0.33895 | 1.02E-14 | 7.54E-12 |
| *ZNF646* | -0.33678 | 1.54E-14 | 1.06E-11 |
| *SLC12A7* | -0.33336 | 2.92E-14 | 1.84E-11 |
| *TNRC18* | -0.33181 | 3.91E-14 | 2.30E-11 |
| *PLXNA1* | -0.3277 | 8.35E-14 | 4.38E-11 |
| *ZNF618* | -0.32276 | 2.05E-13 | 9.10E-11 |
| *MCM3AP* | -0.31558 | 7.32E-13 | 2.73E-10 |
| *SNX33* | -0.31553 | 7.38E-13 | 2.73E-10 |
| *TECPR1* | -0.31007 | 1.90E-12 | 6.04E-10 |
| *ZFP41* | -0.30955 | 2.08E-12 | 6.50E-10 |
| *ZNF496* | -0.30912 | 2.24E-12 | 6.88E-10 |
| *WDR81* | -0.30288 | 6.42E-12 | 1.81E-09 |
| *MLLT6* | -0.30224 | 7.14E-12 | 1.98E-09 |
| *LAMA5* | -0.3018 | 7.69E-12 | 2.11E-09 |
| *SETD1B* | -0.3001 | 1.02E-11 | 2.72E-09 |
| *IQSEC1* | -0.29506 | 2.32E-11 | 5.66E-09 |
| *NCOR2* | -0.29409 | 2.72E-11 | 6.47E-09 |
| *PLEC* | -0.29368 | 2.90E-11 | 6.83E-09 |
| *CREBBP* | -0.29333 | 3.07E-11 | 7.14E-09 |
| *TRRAP* | -0.29255 | 3.48E-11 | 7.71E-09 |
| *EHMT1* | -0.29251 | 3.50E-11 | 7.71E-09 |
| *ARAP1* | -0.2925 | 3.51E-11 | 7.71E-09 |
| *PKHD1* | -0.28995 | 5.27E-11 | 1.09E-08 |
| *PHLPP1* | -0.28994 | 5.28E-11 | 1.09E-08 |
| *MAB21L3* | -0.28924 | 5.90E-11 | 1.19E-08 |
| *MICAL3* | -0.28875 | 6.37E-11 | 1.27E-08 |
| *CABIN1* | -0.28794 | 7.24E-11 | 1.43E-08 |
| *IL17RA* | -0.28537 | 1.08E-10 | 2.02E-08 |
| *MAML1* | -0.28404 | 1.33E-10 | 2.34E-08 |
| *POLE* | -0.28277 | 1.62E-10 | 2.80E-08 |
| *PRAG1* | -0.28181 | 1.88E-10 | 3.21E-08 |
| *IRAK1* | -0.28136 | 2.01E-10 | 3.41E-08 |
| *CLIP2* | -0.28093 | 2.15E-10 | 3.58E-08 |
| *NUP62* | -0.27992 | 2.51E-10 | 4.08E-08 |
| *SUN2* | -0.27803 | 3.35E-10 | 5.03E-08 |
| *TBC1D16* | -0.27633 | 4.32E-10 | 6.06E-08 |
| *ZNF407* | -0.27631 | 4.33E-10 | 6.06E-08 |
| *SLC4A2* | -0.27546 | 4.92E-10 | 6.79E-08 |
| *LINGO3* | -0.27524 | 5.09E-10 | 6.98E-08 |
| *ABCA3* | -0.27411 | 6.03E-10 | 7.98E-08 |
| *PKD1* | -0.27309 | 7.02E-10 | 8.94E-08 |
| *CDK13* | -0.27199 | 8.25E-10 | 1.03E-07 |
| *MCM5* | -0.27151 | 8.86E-10 | 1.10E-07 |
| *AGFG2* | -0.26993 | 1.12E-09 | 1.35E-07 |
| *TRIM56* | -0.26943 | 1.20E-09 | 1.41E-07 |
| *ARHGEF37* | -0.26841 | 1.40E-09 | 1.61E-07 |
| *RAPGEF5* | -0.26822 | 1.43E-09 | 1.65E-07 |
| *GCN1* | -0.26815 | 1.45E-09 | 1.66E-07 |
| *SRCAP* | -0.26759 | 1.57E-09 | 1.79E-07 |
| *TSHZ1* | -0.26717 | 1.67E-09 | 1.88E-07 |
| *TCF20* | -0.26661 | 1.81E-09 | 2.02E-07 |
| *DOCK6* | -0.26646 | 1.85E-09 | 2.06E-07 |
| *ZZEF1* | -0.26616 | 1.93E-09 | 2.13E-07 |
| *ZNF609* | -0.26546 | 2.14E-09 | 2.31E-07 |
| *BAHCC1* | -0.26483 | 2.34E-09 | 2.49E-07 |
| *GIGYF1* | -0.26449 | 2.46E-09 | 2.59E-07 |
| *FOXK1* | -0.26443 | 2.48E-09 | 2.60E-07 |
| *TSC2* | -0.26437 | 2.50E-09 | 2.60E-07 |
| *ZSWIM4* | -0.26416 | 2.58E-09 | 2.67E-07 |
| *PTPDC1* | -0.26334 | 2.90E-09 | 2.95E-07 |
| *JARID2* | -0.26236 | 3.33E-09 | 3.33E-07 |
| *C2CD3* | -0.26217 | 3.42E-09 | 3.39E-07 |
| *CASS4* | -0.26191 | 3.55E-09 | 3.46E-07 |
| *INTS1* | -0.2616 | 3.71E-09 | 3.60E-07 |
| *MEGF6* | -0.26157 | 3.72E-09 | 3.60E-07 |
| *PLXNB2* | -0.26117 | 3.94E-09 | 3.70E-07 |
| *RNF44* | -0.26081 | 4.15E-09 | 3.86E-07 |
| *SMCR8* | -0.26072 | 4.20E-09 | 3.89E-07 |
| *PRDM15* | -0.26003 | 4.63E-09 | 4.23E-07 |
| *ACSL5* | -0.25993 | 4.69E-09 | 4.27E-07 |
| *TYK2* | -0.25909 | 5.28E-09 | 4.78E-07 |
| *TCP11L2* | -0.25844 | 5.78E-09 | 5.08E-07 |
| *NUTM2D* | -0.25825 | 5.93E-09 | 5.16E-07 |
| *VAV2* | -0.25765 | 6.46E-09 | 5.59E-07 |
| *URB1* | -0.2573 | 6.77E-09 | 5.84E-07 |
| *JADE2* | -0.25592 | 8.20E-09 | 6.83E-07 |
| *ARID3A* | -0.25512 | 9.16E-09 | 7.51E-07 |
| *IRS1* | -0.25481 | 9.56E-09 | 7.77E-07 |
| *CRAMP1* | -0.25465 | 9.77E-09 | 7.88E-07 |
| *TCP11L1* | -0.25417 | 1.04E-08 | 8.39E-07 |
| *RAI1* | -0.25412 | 1.05E-08 | 8.41E-07 |
| *GEMIN5* | -0.2538 | 1.10E-08 | 8.75E-07 |
| *SIPA1L3* | -0.25377 | 1.10E-08 | 8.75E-07 |
| *RAP1GAP2* | -0.25358 | 1.13E-08 | 8.91E-07 |
| *IP6K1* | -0.25338 | 1.16E-08 | 9.08E-07 |
| *KMT2B* | -0.25153 | 1.50E-08 | 1.12E-06 |
| *ACVR2B* | -0.25146 | 1.51E-08 | 1.13E-06 |
| *WDR24* | -0.25037 | 1.75E-08 | 1.29E-06 |
| *NEU3* | -0.24956 | 1.95E-08 | 1.4E-06 |
| *WWC1* | -0.24917 | 2.06E-08 | 1.46E-06 |
| *ZNF862* | -0.24891 | 2.13E-08 | 1.5E-06 |
| *MYH9* | -0.24867 | 2.20E-08 | 1.54E-06 |
| *ZSCAN25* | -0.24813 | 2.36E-08 | 1.65E-06 |
| *NEK6* | -0.24769 | 2.51E-08 | 1.74E-06 |
| *TNFSF15* | -0.24706 | 2.73E-08 | 1.88E-06 |
| *WSCD1* | -0.24655 | 2.92E-08 | 1.98E-06 |
| *CARD10* | -0.24641 | 2.97E-08 | 2.01E-06 |
| *RBM33* | -0.24617 | 3.07E-08 | 2.06E-06 |
| *BAIAP3* | -0.24607 | 3.11E-08 | 2.07E-06 |
| *ZNF74* | -0.24575 | 3.25E-08 | 2.14E-06 |
| *RRP1B* | -0.24534 | 3.43E-08 | 2.23E-06 |
| *KMT2D* | -0.24533 | 3.43E-08 | 2.23E-06 |
| *ZNF710* | -0.24524 | 3.47E-08 | 2.25E-06 |
| *DNAJC22* | -0.24453 | 3.81E-08 | 2.44E-06 |
| *GGA3* | -0.24442 | 3.87E-08 | 2.47E-06 |
| *SVEP1* | -0.24424 | 3.96E-08 | 2.52E-06 |
| *LIMD1* | -0.24419 | 3.98E-08 | 2.52E-06 |
| *KIAA1549* | -0.24416 | 4.00E-08 | 2.52E-06 |
| *TONSL* | -0.24398 | 4.10E-08 | 2.57E-06 |
| *ZMIZ1* | -0.24382 | 4.18E-08 | 2.61E-06 |
| *CASKIN1* | -0.24371 | 4.25E-08 | 2.64E-06 |
| *RASGEF1B* | -0.24357 | 4.32E-08 | 2.68E-06 |
| *MEGF8* | -0.24341 | 4.41E-08 | 2.72E-06 |
| *INCENP* | -0.24331 | 4.47E-08 | 2.73E-06 |
| *ARMC9* | -0.24269 | 4.85E-08 | 2.93E-06 |
| *CASTOR3* | -0.24252 | 4.96E-08 | 2.97E-06 |
| *USP49* | -0.24246 | 5.00E-08 | 2.98E-06 |
| *NUP214* | -0.24241 | 5.03E-08 | 2.99E-06 |
| *PTPN3* | -0.24199 | 5.31E-08 | 3.12E-06 |
| *TBC1D2B* | -0.24186 | 5.41E-08 | 3.14E-06 |
| *ALPK2* | -0.24176 | 5.47E-08 | 3.15E-06 |
| *NSD2* | -0.24174 | 5.49E-08 | 3.15E-06 |
| *SMYD4* | -0.24161 | 5.58E-08 | 3.18E-06 |
| *RPH3AL* | -0.24113 | 5.94E-08 | 3.36E-06 |
| *EPG5* | -0.24087 | 6.15E-08 | 3.44E-06 |
| *ZNF608* | -0.24085 | 6.16E-08 | 3.44E-06 |
| *KMT2C* | -0.24016 | 6.74E-08 | 3.73E-06 |
| *PLEKHA7* | -0.23984 | 7.02E-08 | 3.86E-06 |
| *SBF1* | -0.23962 | 7.23E-08 | 3.94E-06 |
| *NOTUM* | -0.23947 | 7.36E-08 | 3.99E-06 |
| *SETDB1* | -0.23911 | 7.71E-08 | 4.15E-06 |
| *CNKSR2* | -0.23808 | 8.80E-08 | 4.67E-06 |
| *SLC12A9* | -0.23778 | 9.14E-08 | 4.81E-06 |
| *AP5Z1* | -0.23758 | 9.38E-08 | 4.89E-06 |
| *CCDC97* | -0.23749 | 9.48E-08 | 4.92E-06 |
| *CPSF1* | -0.23738 | 9.62E-08 | 4.96E-06 |
| *LAT2* | -0.23697 | 1.01E-07 | 5.21E-06 |
| *LRP5* | -0.23665 | 1.06E-07 | 5.42E-06 |
| *PDPK1* | -0.23653 | 1.07E-07 | 5.48E-06 |
| *ARHGAP35* | -0.23646 | 1.08E-07 | 5.52E-06 |
| *SEMA6C* | -0.23617 | 1.12E-07 | 5.7E-06 |
| *F2RL2* | -0.23571 | 1.19E-07 | 6E-06 |
| *SRRM2* | -0.23558 | 1.21E-07 | 6.09E-06 |
| *HIP1* | -0.23534 | 1.25E-07 | 6.26E-06 |
| *PDCD11* | -0.23514 | 1.28E-07 | 6.41E-06 |
| *H6PD* | -0.23505 | 1.29E-07 | 6.45E-06 |
| *RNF213* | -0.23464 | 1.36E-07 | 6.71E-06 |
| *NEURL2* | -0.23434 | 1.41E-07 | 6.93E-06 |
| *MAPKBP1* | -0.23369 | 1.53E-07 | 7.41E-06 |
| *FRMD4A* | -0.23366 | 1.54E-07 | 7.42E-06 |
| *PCNT* | -0.23352 | 1.57E-07 | 7.52E-06 |
| *SCARF2* | -0.23325 | 1.62E-07 | 7.7E-06 |
| *POM121C* | -0.23317 | 1.64E-07 | 7.76E-06 |
| *PLD4* | -0.23254 | 1.77E-07 | 8.32E-06 |
| *BMF* | -0.23192 | 1.91E-07 | 8.85E-06 |
| *ABCA2* | -0.23191 | 1.92E-07 | 8.85E-06 |
| *SOCS7* | -0.23182 | 1.94E-07 | 8.91E-06 |
| *POM121* | -0.23157 | 2.00E-07 | 9.15E-06 |
| *FBXL18* | -0.23153 | 2.01E-07 | 9.17E-06 |
| *ZNF740* | -0.23103 | 2.14E-07 | 9.64E-06 |
| *ZMYND8* | -0.231 | 2.15E-07 | 9.65E-06 |
| *NR2F1* | -0.23083 | 2.19E-07 | 9.82E-06 |
| *SHANK2* | -0.23065 | 2.24E-07 | 1E-05 |
| *AMER1* | -0.23051 | 2.28E-07 | 1.02E-05 |
| *ZBTB16* | -0.2305 | 2.28E-07 | 1.02E-05 |
| *MNT* | -0.23048 | 2.29E-07 | 1.02E-05 |
| *MAU2* | -0.23021 | 2.37E-07 | 1.04E-05 |
| *ISLR2* | -0.22953 | 2.57E-07 | 1.12E-05 |
| *SETD1A* | -0.22947 | 2.59E-07 | 1.13E-05 |
| *KDM2B* | -0.22934 | 2.63E-07 | 1.14E-05 |
| *RPTOR* | -0.22927 | 2.66E-07 | 1.15E-05 |
| *TMEM94* | -0.22907 | 2.72E-07 | 1.18E-05 |
| *SMG1P3* | -0.22873 | 2.84E-07 | 1.22E-05 |
| *ANKHD1-EIF4EBP3* | -0.22844 | 2.94E-07 | 1.26E-05 |
| *IGFBP5* | -0.22826 | 3.01E-07 | 1.28E-05 |
| *C5* | -0.22815 | 3.05E-07 | 1.29E-05 |
| *FOXO4* | -0.22783 | 3.17E-07 | 1.34E-05 |
| *ZNF623* | -0.22754 | 3.28E-07 | 1.38E-05 |
| *MINDY1* | -0.22743 | 3.32E-07 | 1.39E-05 |
| *NFATC2IP* | -0.22731 | 3.38E-07 | 1.41E-05 |
| *UBAP2* | -0.2273 | 3.38E-07 | 1.41E-05 |
| *FAM78B* | -0.2273 | 3.38E-07 | 1.41E-05 |
| *MLXIP* | -0.22724 | 3.40E-07 | 1.41E-05 |
| *ZNF169* | -0.22688 | 3.56E-07 | 1.46E-05 |
| *PDZD2* | -0.22656 | 3.70E-07 | 1.51E-05 |
| *MBP* | -0.22614 | 3.89E-07 | 1.58E-05 |
| *TPCN1* | -0.22603 | 3.94E-07 | 1.59E-05 |
| *TTLL12* | -0.226 | 3.96E-07 | 1.59E-05 |
| *CPZ* | -0.22599 | 3.96E-07 | 1.59E-05 |
| *ABR* | -0.22584 | 4.04E-07 | 1.62E-05 |
| *LLGL1* | -0.22575 | 4.08E-07 | 1.63E-05 |
| *MYO9B* | -0.22572 | 4.09E-07 | 1.63E-05 |
| *ARHGAP26* | -0.22564 | 4.13E-07 | 1.64E-05 |
| *RNF216* | -0.22561 | 4.15E-07 | 1.64E-05 |
| *MYO18A* | -0.22544 | 4.24E-07 | 1.67E-05 |
