# Supplementary material for: PACT is requisite for prostate cancer cell proliferation
Source: Sci Rep. 2025 Oct 21;15:36610. doi: 10.1038/s41598-025-20494-9 (PMC12540807; doi:10.1038/s41598-025-20494-9)
Supplement: Supplementary file 5 — Supplementary Material 5 [file 41598_2025_20494_MOESM5_ESM.docx]

**Supplementary Table 3.** Downregulated genes in the PACT KO cells as compared to parental LNCaP. The genes validated and further investigated herein are in bold and are namely: *H2AFJ* (H2A histone family member J)*, PSMD5* (proteasome 26s non-ATPase subunit, 5)*, AQP3* (Aquaporin 3), *TMEM45B* (transmembrane protein 45B), *SLC22A3* (Solute carrier family 22 member 3), *KLK3* (Kallikrein related peptidase 3, Prostate specific antigen, PSA), and *KLK2* (Human kallikrein), and were respectively downregulated 10.76, 7.18, 5.88, 4.63, 3.9, 2.49, 0.83 log_2_ fold change in the PACT KO cells.

| **Gene Name** | **Log_2_ FC** | **P Value** | **FDR** |
| --- | --- | --- | --- |
| ***H2AFJ*** | **10.758** | **1.33E-44** | **1.36E-41** |
| *LXN* | 8.474 | 2.37E-20 | 1.08E-18 |
| *THBS1* | 7.619 | 2.63E-30 | 3.82E-28 |
| *FCAMR* | 7.531 | 5.54E-13 | 9.27E-12 |
| *ADRB2* | 7.208 | 8.73E-11 | 1.06E-09 |
| ***PSMD5*** | **7.179** | **1.67E-33** | **3.39E-31** |
| *EFS* | 7.162 | 7.05E-34 | 1.45E-31 |
| *SEMA6D* | 7.123 | 2.16E-10 | 2.49E-09 |
| ***AQP3*** | **5.878** | **3.09E-31** | **5.06E-29** |
| *SLC45A1* | 5.699 | 4.96E-26 | 4.44E-24 |
| *RBP7* | 5.537 | 7.68E-17 | 2.17E-15 |
| *DSC3* | 5.341 | 6.69E-15 | 1.45E-13 |
| *CES4A* | 5.230 | 5.40E-14 | 1.05E-12 |
| *MT1G* | 5.065 | 2.48E-16 | 6.57E-15 |
| *ZNF785* | 4.888 | 3.50E-11 | 4.47E-10 |
| ***TMEM45B*** | **4.626** | **5.06E-36** | **1.47E-33** |
| *CDH3* | 4.540 | 1.98E-09 | 1.93E-08 |
| *STAT5A* | 4.530 | 1.21E-16 | 3.32E-15 |
| *PSTPIP2* | 4.422 | 2.31E-11 | 3.04E-10 |
| *ERVMER34-1* | 4.297 | 6.23E-21 | 3.03E-19 |
| *CTAG2* | 4.112 | 3.53E-09 | 3.31E-08 |
| *CCDC74B* | 4.061 | 5.08E-09 | 4.60E-08 |
| *PCDH11X* | 4.061 | 7.29E-09 | 6.43E-08 |
| *FCHSD1* | 4.035 | 5.63E-09 | 5.07E-08 |
| ***SLC22A3*** | **3.945** | **8.63E-33** | **1.62E-30** |
| *PCDH11Y* | 3.918 | 8.57E-23 | 5.06E-21 |
| *CYP1A1* | 3.505 | 3.24E-15 | 7.31E-14 |
| *POF1B* | 3.455 | 4.87E-20 | 2.11E-18 |
| *PTPN20* | 3.299 | 2.65E-24 | 1.89E-22 |
| *SMIM3* | 3.218 | 2.81E-08 | 2.26E-07 |
| *SMIM10L2A* | 3.168 | 4.83E-13 | 8.17E-12 |
| *WDR76* | 3.163 | 3.30E-34 | 7.59E-32 |
| *GPLD1* | 3.140 | 1.05E-07 | 7.68E-07 |
| *RGS16* | 3.028 | 8.01E-09 | 7.01E-08 |
| *MAGEC1* | 3.003 | 1.18E-13 | 2.19E-12 |
| *MECOM* | 2.824 | 9.41E-14 | 1.76E-12 |
| *RIPOR2* | 2.690 | 6.54E-21 | 3.16E-19 |
| *TWIST1* | 2.684 | 1.85E-07 | 1.31E-06 |
| *PLP2* | 2.562 | 1.20E-19 | 4.96E-18 |
| ***KLK3*** | **2.495** | **2.97E-61** | **1.41E-57** |
| *HENMT1* | 2.453 | 2.60E-29 | 3.42E-27 |
| *UTY* | 2.412 | 3.41E-11 | 4.35E-10 |
| *TUSC1* | 2.388 | 6.00E-12 | 8.62E-11 |
| *TLL1* | 2.348 | 7.76E-27 | 7.89E-25 |
| *PHTF1* | 2.341 | 1.32E-09 | 1.35E-08 |
| *BHLHA15* | 2.260 | 2.51E-06 | 1.43E-05 |
| *PRXL2A* | 2.218 | 7.53E-21 | 3.57E-19 |
| *MAPK13* | 2.197 | 2.86E-19 | 1.13E-17 |
| *ALDH5A1* | 2.134 | 1.45E-24 | 1.10E-22 |
| *CBLC* | 2.088 | 4.91E-24 | 3.36E-22 |
| *RGS11* | 2.085 | 6.83E-15 | 1.48E-13 |
| *ESYT3* | 2.064 | 8.83E-11 | 1.07E-09 |
| *EVX1* | 2.052 | 3.01E-05 | 0.000137 |
| *AHR* | 2.029 | 5.99E-08 | 4.56E-07 |
| *DPP4* | 1.977 | 8.63E-07 | 5.35E-06 |
| *HS6ST2* | 1.932 | 6.35E-23 | 3.86E-21 |
| *SLC5A1* | 1.929 | 8.75E-06 | 4.48E-05 |
| *CLIP4* | 1.921 | 3.29E-13 | 5.71E-12 |
| *HOXC10* | 1.905 | 3.30E-18 | 1.13E-16 |
| *RGS17* | 1.871 | 1.27E-06 | 7.64E-06 |
| *TUBB6* | 1.867 | 5.74E-07 | 3.70E-06 |
| *OBSL1* | 1.860 | 0.000112 | 0.000452 |
| *ABCG2* | 1.819 | 4.96E-08 | 3.84E-07 |
| *PARP10* | 1.806 | 6.96E-42 | 4.31E-39 |
| *TRERF1* | 1.772 | 3.95E-36 | 1.20E-33 |
| *TMSB4X* | 1.738 | 1.01E-18 | 3.72E-17 |
| *SLC6A17* | 1.728 | 3.49E-05 | 0.000156 |
| *TRIM58* | 1.718 | 8.53E-05 | 0.000353 |
| *S100P* | 1.666 | 0.000137 | 0.000542 |
| *FNDC4* | 1.656 | 2.84E-09 | 2.70E-08 |
| *FADS2* | 1.655 | 1.78E-73 | 2.53E-69 |
| *CADPS2* | 1.629 | 7.66E-11 | 9.38E-10 |
| *SLC39A8* | 1.628 | 4.43E-44 | 4.20E-41 |
| *FOXN4* | 1.618 | 2.65E-11 | 3.46E-10 |
| *TGFBR2* | 1.618 | 1.73E-06 | 1.01E-05 |
| *GLYATL2* | 1.615 | 1.17E-06 | 7.10E-06 |
| *GUCY1A2* | 1.586 | 9.08E-06 | 4.64E-05 |
| *CLEC7A* | 1.580 | 8.07E-12 | 1.14E-10 |
| *SYTL2* | 1.568 | 2.12E-26 | 1.98E-24 |
| *STAB1* | 1.567 | 4.28E-05 | 0.000189 |
| *FZD2* | 1.565 | 2.03E-08 | 1.67E-07 |
| *PRKRA* | 1.563 | 1.05E-39 | 4.83E-37 |
| *SHC3* | 1.562 | 3.15E-11 | 4.05E-10 |
| *ANK1* | 1.536 | 0.000367 | 0.001318 |
| *NCKAP5* | 1.527 | 4.14E-07 | 2.73E-06 |
| *ACSS1* | 1.523 | 6.79E-09 | 6.02E-08 |
| *KIAA1324* | 1.521 | 1.32E-17 | 4.16E-16 |
| *PPP1R14C* | 1.514 | 1.70E-21 | 8.68E-20 |
| *B4GALNT1* | 1.512 | 5.54E-31 | 8.86E-29 |
| *CLU* | 1.506 | 3.66E-27 | 3.80E-25 |
| *NIBAN1* | 1.504 | 2.22E-22 | 1.25E-20 |
| *PLIN2* | 1.501 | 1.72E-05 | 8.25E-05 |
| *CD163L1* | 1.499 | 2.56E-15 | 5.88E-14 |
| *KLHL3* | 1.490 | 2.92E-10 | 3.31E-09 |
| *SWAP70* | 1.457 | 1.75E-23 | 1.12E-21 |
| *UGT2B10* | 1.448 | 9.57E-26 | 8.30E-24 |
| *SYCP2L* | 1.426 | 0.003001 | 0.008544 |
| *DAPK1* | 1.419 | 2.76E-22 | 1.53E-20 |
| *SLC34A3* | 1.415 | 1.08E-07 | 7.93E-07 |
| *SFN* | 1.409 | 3.08E-05 | 0.00014 |
| *GULP1* | 1.405 | 6.61E-58 | 1.88E-54 |
| *LHX6* | 1.396 | 2.05E-06 | 1.18E-05 |
| *GPX2* | 1.383 | 2.24E-05 | 0.000105 |
| *LACC1* | 1.374 | 1.91E-09 | 1.88E-08 |
| *MCUB* | 1.372 | 4.25E-06 | 2.32E-05 |
| *PABPC5* | 1.368 | 3.29E-16 | 8.49E-15 |
| *H2BC8* | 1.348 | 0.000166 | 0.000646 |
| *KCNH8* | 1.338 | 0.000818 | 0.002699 |
| *SPATA18* | 1.318 | 3.44E-22 | 1.89E-20 |
| *RIT2* | 1.317 | 0.002477 | 0.007241 |
| *SLC23A1* | 1.311 | 8.79E-05 | 0.000362 |
| *SYT4* | 1.305 | 8.30E-38 | 2.88E-35 |
| *SLC16A14* | 1.303 | 6.00E-30 | 8.37E-28 |
| *MALL* | 1.293 | 1.30E-06 | 7.80E-06 |
| *ANO9* | 1.290 | 1.32E-09 | 1.35E-08 |
| *TAFA4* | 1.285 | 8.55E-06 | 4.39E-05 |
| *EVA1B* | 1.285 | 1.63E-15 | 3.87E-14 |
| *BMPR1B* | 1.262 | 7.52E-44 | 6.69E-41 |
| *TNFRSF14* | 1.250 | 3.02E-07 | 2.06E-06 |
| *MAP1LC3A* | 1.241 | 1.39E-14 | 2.91E-13 |
| *FLT4* | 1.229 | 8.74E-07 | 5.42E-06 |
| *TERT* | 1.215 | 3.41E-09 | 3.20E-08 |
| *SPRY1* | 1.205 | 1.46E-12 | 2.29E-11 |
| *LHX9* | 1.188 | 3.17E-05 | 0.000143 |
| *ZFAND4* | 1.186 | 3.85E-07 | 2.56E-06 |
| *SLC13A3* | 1.176 | 8.35E-06 | 4.29E-05 |
| *GNA14* | 1.163 | 6.27E-05 | 0.000267 |
| *FN3K* | 1.160 | 1.88E-12 | 2.91E-11 |
| *TM4SF1* | 1.146 | 1.55E-07 | 1.11E-06 |
| *FERMT1* | 1.130 | 1.73E-14 | 3.58E-13 |
| *GGT1* | 1.129 | 7.67E-14 | 1.46E-12 |
| *DGKH* | 1.128 | 1.31E-27 | 1.42E-25 |
| *ACTRT3* | 1.124 | 2.55E-18 | 8.79E-17 |
| *TMPRSS11E* | 1.120 | 4.73E-07 | 3.09E-06 |
| *H2AC8* | 1.116 | 1.35E-06 | 8.09E-06 |
| *CCDC122* | 1.115 | 8.00E-05 | 0.000333 |
| *PHLDA2* | 1.113 | 2.13E-19 | 8.50E-18 |
| *CNGA1* | 1.108 | 1.85E-09 | 1.83E-08 |
| *MNX1* | 1.106 | 3.64E-09 | 3.39E-08 |
| *ATF5* | 1.101 | 4.46E-42 | 2.88E-39 |
| *ZNF594* | 1.101 | 0.000558 | 0.001915 |
| *EEF1A2* | 1.087 | 2.82E-57 | 6.69E-54 |
| *PTK6* | 1.086 | 7.13E-12 | 1.02E-10 |
| *PRTG* | 1.083 | 8.20E-16 | 2.01E-14 |
| *PRUNE2* | 1.082 | 1.44E-59 | 5.14E-56 |
| *FAXDC2* | 1.068 | 0.001353 | 0.004243 |
| *CTNNA3* | 1.067 | 2.30E-09 | 2.22E-08 |
| *HES4* | 1.059 | 6.31E-08 | 4.79E-07 |
| *ZFHX4* | 1.043 | 6.51E-14 | 1.25E-12 |
| *HTATIP2* | 1.042 | 6.96E-09 | 6.16E-08 |
| *CADM1* | 1.041 | 9.54E-14 | 1.78E-12 |
| *AGR2* | 1.032 | 0.000125 | 0.000498 |
| *GNG3* | 1.032 | 0.00286 | 0.008193 |
| *BMP8B* | 1.028 | 7.16E-06 | 3.72E-05 |
| *SCARA3* | 1.020 | 9.52E-10 | 9.93E-09 |
| *CTSC* | 1.018 | 3.75E-12 | 5.58E-11 |
| *UGT2B28* | 1.011 | 0.001281 | 0.004048 |
| *SP140L* | 0.988 | 0.000921 | 0.003011 |
| *CRYBG1* | 0.981 | 4.58E-11 | 5.75E-10 |
| *SLC7A5* | 0.973 | 8.89E-16 | 2.17E-14 |
| *TMEM205* | 0.970 | 2.27E-42 | 1.61E-39 |
| *ZNF711* | 0.968 | 8.76E-12 | 1.23E-10 |
| *CRYBG2* | 0.956 | 1.18E-05 | 5.88E-05 |
| *PDZK1IP1* | 0.946 | 8.44E-05 | 0.00035 |
| *MT2A* | 0.939 | 1.90E-30 | 2.82E-28 |
| *UGT2B11* | 0.931 | 3.42E-20 | 1.50E-18 |
| *ZNF665* | 0.927 | 0.001672 | 0.005123 |
| *APP* | 0.920 | 2.12E-52 | 3.78E-49 |
| *NFU1* | 0.920 | 2.54E-23 | 1.61E-21 |
| *LCP1* | 0.919 | 5.28E-28 | 6.02E-26 |
| *ASPHD1* | 0.910 | 3.51E-16 | 8.97E-15 |
| *TMEM165* | 0.905 | 5.13E-35 | 1.33E-32 |
| *ITPR2* | 0.902 | 4.12E-12 | 6.10E-11 |
| *COX7B2* | 0.900 | 1.59E-07 | 1.13E-06 |
| *TMEM179* | 0.897 | 1.08E-05 | 5.44E-05 |
| *ANKAR* | 0.891 | 6.54E-05 | 0.000277 |
| *HSD17B12* | 0.891 | 5.62E-35 | 1.40E-32 |
| *PMP22* | 0.890 | 0.000954 | 0.003106 |
| *NOS3* | 0.884 | 0.002412 | 0.007069 |
| *ALS2CL* | 0.876 | 2.74E-11 | 3.56E-10 |
| *GJB1* | 0.867 | 2.03E-07 | 1.43E-06 |
| *FADS1* | 0.857 | 1.70E-36 | 5.38E-34 |
| *UGT2B15* | 0.853 | 6.21E-41 | 3.40E-38 |
| *SMAGP* | 0.852 | 0.000555 | 0.001906 |
| *SLC1A3* | 0.849 | 4.88E-05 | 0.000213 |
| ***KLK2*** | **0.837** | **2.55E-20** | **1.14E-18** |
| *UGT2B17* | 0.834 | 7.18E-35 | 1.73E-32 |
| *HMGCS2* | 0.827 | 4.15E-15 | 9.25E-14 |
| *SEMA4G* | 0.826 | 1.24E-09 | 1.28E-08 |
| *SPC25* | 0.826 | 2.40E-12 | 3.68E-11 |
| *CBR3* | 0.821 | 2.60E-07 | 1.79E-06 |
| *PAK3* | 0.816 | 0.000929 | 0.003033 |
| *MYBL2* | 0.811 | 1.94E-34 | 4.53E-32 |
| *FAM72D* | 0.803 | 0.000658 | 0.002222 |
| *OSBPL10* | 0.802 | 3.11E-05 | 0.000141 |
| *RASD1* | 0.801 | 6.06E-06 | 3.21E-05 |
| *PLK1* | 0.801 | 4.32E-31 | 7.00E-29 |
| *IL36RN* | 0.793 | 0.000251 | 0.000933 |
| *GADD45B* | 0.791 | 5.08E-06 | 2.73E-05 |
| *PKMYT1* | 0.790 | 2.23E-16 | 5.99E-15 |
| *THRB* | 0.777 | 2.71E-26 | 2.49E-24 |
| *ZNF185* | 0.775 | 4.30E-08 | 3.36E-07 |
| *SHCBP1* | 0.774 | 1.82E-18 | 6.49E-17 |
| *RRM2* | 0.773 | 4.22E-35 | 1.13E-32 |
| *TRIM46* | 0.773 | 9.87E-06 | 5.01E-05 |
| *IGFBP2* | 0.772 | 5.63E-28 | 6.31E-26 |
| *EXO1* | 0.769 | 2.09E-15 | 4.85E-14 |
| *AADAT* | 0.767 | 1.67E-22 | 9.55E-21 |
| *RPP25* | 0.767 | 0.000984 | 0.003194 |
| *ADRA2A* | 0.766 | 3.34E-07 | 2.24E-06 |
| *EPHX4* | 0.766 | 6.49E-06 | 3.42E-05 |
| *H1-2* | 0.761 | 5.78E-34 | 1.25E-31 |
| *ISYNA1* | 0.761 | 1.75E-13 | 3.16E-12 |
| *RCN3* | 0.757 | 3.02E-07 | 2.06E-06 |
| *FANCA* | 0.757 | 7.55E-22 | 4.01E-20 |
| *KIFC1* | 0.752 | 1.03E-25 | 8.79E-24 |
| *ASTN1* | 0.749 | 0.000117 | 0.00047 |
| *SDC1* | 0.749 | 6.41E-29 | 8.08E-27 |
| *SLITRK6* | 0.748 | 5.32E-05 | 0.00023 |
| *ADA2* | 0.742 | 0.000854 | 0.002807 |
| *HOXC12* | 0.742 | 0.000959 | 0.00312 |
| *CARMIL2* | 0.741 | 0.001367 | 0.00428 |
| *REEP2* | 0.737 | 2.04E-07 | 1.43E-06 |
| *NAV3* | 0.729 | 1.05E-06 | 6.41E-06 |
| *ESPL1* | 0.729 | 1.50E-23 | 9.71E-22 |
| *CENPA* | 0.720 | 7.36E-10 | 7.80E-09 |
| *PLEKHF1* | 0.719 | 7.38E-05 | 0.00031 |
| *OIP5* | 0.715 | 1.36E-12 | 2.15E-11 |
| *VWA8* | 0.715 | 6.70E-21 | 3.21E-19 |
| *TYMS* | 0.713 | 4.67E-33 | 9.11E-31 |
| *PIF1* | 0.712 | 1.77E-10 | 2.06E-09 |
| *TCF19* | 0.707 | 2.02E-24 | 1.46E-22 |
| *SKA3* | 0.705 | 7.60E-12 | 1.08E-10 |
| *ESCO2* | 0.702 | 4.00E-18 | 1.35E-16 |
| *RBM24* | 0.701 | 2.09E-09 | 2.03E-08 |
| *UHRF1* | 0.701 | 2.55E-16 | 6.74E-15 |
| *EMP2* | 0.700 | 5.09E-17 | 1.48E-15 |
| *FAM72B* | 0.699 | 3.14E-06 | 1.76E-05 |
| *KIF2C* | 0.699 | 1.85E-26 | 1.78E-24 |
| *PRIM1* | 0.697 | 1.90E-14 | 3.90E-13 |
| *PEG3* | 0.695 | 3.48E-12 | 5.21E-11 |
| *E2F8* | 0.692 | 3.78E-10 | 4.19E-09 |
| *ASPH* | 0.691 | 1.47E-38 | 5.64E-36 |
| *DUSP27* | 0.687 | 1.22E-05 | 6.05E-05 |
| *NEK2* | 0.687 | 8.30E-17 | 2.32E-15 |
| *CKAP2L* | 0.686 | 5.55E-15 | 1.22E-13 |
| *CENPI* | 0.686 | 5.04E-13 | 8.51E-12 |
| *TICRR* | 0.684 | 7.22E-16 | 1.79E-14 |
| *RASEF* | 0.684 | 1.91E-28 | 2.31E-26 |
| *BLM* | 0.684 | 2.93E-10 | 3.31E-09 |
| *PAQR4* | 0.682 | 2.81E-14 | 5.65E-13 |
| *MTFR2* | 0.682 | 1.24E-06 | 7.48E-06 |
| *ASF1B* | 0.682 | 1.60E-19 | 6.55E-18 |
| *GRIN2C* | 0.681 | 0.00031 | 0.001129 |
| *TACC3* | 0.681 | 4.68E-35 | 1.23E-32 |
| *FH* | 0.681 | 4.68E-36 | 1.39E-33 |
| *DNAH8* | 0.680 | 0.000528 | 0.001826 |
| *HYAL3* | 0.680 | 4.45E-16 | 1.13E-14 |
| *TEAD4* | 0.677 | 4.35E-10 | 4.80E-09 |
| *DIAPH3* | 0.676 | 4.59E-13 | 7.79E-12 |
| *BRI3BP* | 0.676 | 4.06E-14 | 7.95E-13 |
| *KIF15* | 0.675 | 8.35E-13 | 1.36E-11 |
| *NCAPG* | 0.672 | 5.91E-15 | 1.29E-13 |
| *POLR3G* | 0.671 | 1.54E-09 | 1.56E-08 |
| *SLC29A1* | 0.661 | 2.46E-20 | 1.11E-18 |
| *FTCDNL1* | 0.660 | 0.002569 | 0.007465 |
| *HGD* | 0.658 | 3.12E-12 | 4.70E-11 |
| *OR51E1* | 0.656 | 9.74E-08 | 7.19E-07 |
| *CDCA5* | 0.655 | 8.14E-26 | 7.15E-24 |
| *COL26A1* | 0.655 | 8.27E-06 | 4.25E-05 |
| *EPPK1* | 0.654 | 2.56E-25 | 2.13E-23 |
| *ABCA12* | 0.651 | 1.09E-08 | 9.37E-08 |
| *SLC27A2* | 0.649 | 3.31E-11 | 4.24E-10 |
| *FAM111B* | 0.648 | 1.14E-24 | 8.66E-23 |
| *MT1X* | 0.647 | 8.75E-13 | 1.42E-11 |
| *RAD54L* | 0.647 | 3.94E-17 | 1.17E-15 |
| *DDR2* | 0.647 | 1.33E-07 | 9.67E-07 |
| *OAS3* | 0.646 | 3.07E-14 | 6.14E-13 |
| *TUBA4A* | 0.644 | 9.85E-33 | 1.82E-30 |
| *TNFRSF10C* | 0.644 | 0.000633 | 0.002145 |
| *NQO1* | 0.644 | 1.28E-29 | 1.72E-27 |
| *NUF2* | 0.643 | 1.28E-14 | 2.68E-13 |
| *DNAH2* | 0.642 | 3.06E-07 | 2.08E-06 |
| *NDUFA4L2* | 0.641 | 0.000696 | 0.002335 |
| *PIMREG* | 0.640 | 1.04E-19 | 4.36E-18 |
| *ORC6* | 0.639 | 1.48E-12 | 2.32E-11 |
| *CEBPD* | 0.639 | 0.001126 | 0.00361 |
| *GAL* | 0.637 | 3.01E-09 | 2.85E-08 |
| *GNAI1* | 0.635 | 5.80E-27 | 5.98E-25 |
| *EIF5A2* | 0.633 | 5.18E-07 | 3.36E-06 |
| *MELK* | 0.633 | 1.65E-21 | 8.47E-20 |
| *SLC1A4* | 0.633 | 5.35E-09 | 4.83E-08 |
| *ZNF132* | 0.630 | 3.24E-07 | 2.18E-06 |
| *RECQL4* | 0.629 | 3.19E-21 | 1.58E-19 |
| *FKBP4* | 0.628 | 1.34E-39 | 5.94E-37 |
| *ZNF367* | 0.627 | 2.85E-11 | 3.70E-10 |
| *NXPH4* | 0.626 | 3.49E-13 | 6.04E-12 |
| *MAD2L1* | 0.625 | 6.99E-20 | 3.00E-18 |
| *SLC6A9* | 0.625 | 5.93E-19 | 2.27E-17 |
| *CDCA3* | 0.625 | 6.82E-21 | 3.26E-19 |
| *WDR62* | 0.623 | 4.18E-15 | 9.28E-14 |
| *BIRC5* | 0.622 | 4.66E-29 | 6.04E-27 |
| *KCNK5* | 0.621 | 2.50E-07 | 1.72E-06 |
| *CEP55* | 0.620 | 4.15E-15 | 9.25E-14 |
| *HMMR* | 0.620 | 2.18E-25 | 1.84E-23 |
| *ASRGL1* | 0.618 | 2.41E-06 | 1.37E-05 |
| *CDC20* | 0.617 | 3.44E-28 | 4.01E-26 |
| *WNT5A* | 0.616 | 9.80E-12 | 1.37E-10 |
| *SLC52A3* | 0.615 | 0.000276 | 0.001017 |
| *ADAT3* | 0.615 | 0.00013 | 0.000519 |
| *TROAP* | 0.615 | 3.27E-05 | 0.000147 |
| *TUBA1B* | 0.615 | 6.81E-40 | 3.34E-37 |
| *GINS2* | 0.614 | 4.58E-14 | 8.92E-13 |
| *CDC25C* | 0.614 | 4.74E-09 | 4.32E-08 |
| *PRR11* | 0.612 | 6.43E-20 | 2.78E-18 |
| *ANLN* | 0.612 | 8.78E-25 | 6.80E-23 |
| *FZD8* | 0.610 | 0.001899 | 0.005739 |
| *CDC6* | 0.609 | 2.59E-23 | 1.63E-21 |
| *CCNA2* | 0.608 | 1.24E-20 | 5.79E-19 |
| *DTL* | 0.607 | 9.29E-16 | 2.26E-14 |
| *UAP1* | 0.606 | 3.42E-32 | 6.25E-30 |
| *PARVA* | 0.604 | 6.26E-15 | 1.36E-13 |
| *CDKN3* | 0.604 | 2.15E-14 | 4.39E-13 |
| *VWF* | 0.603 | 8.06E-14 | 1.53E-12 |
| *NCAPH* | 0.603 | 1.05E-19 | 4.40E-18 |
| *PROS1* | 0.601 | 0.001096 | 0.003519 |
| *AC098582.1* | 0.600 | 1.24E-11 | 1.71E-10 |
| *RMI2* | 0.599 | 6.64E-08 | 5.02E-07 |
| *HELLS* | 0.595 | 7.82E-09 | 6.87E-08 |
| *MSH3* | 0.594 | 6.87E-15 | 1.48E-13 |
| *CDC45* | 0.594 | 2.67E-13 | 4.70E-12 |
| *TBC1D4* | 0.593 | 1.46E-17 | 4.57E-16 |
| *MCM10* | 0.592 | 7.27E-17 | 2.06E-15 |
| *PCLAF* | 0.592 | 8.13E-17 | 2.28E-15 |
| *GTSE1* | 0.592 | 5.88E-17 | 1.70E-15 |
| *CHEK2* | 0.589 | 1.04E-12 | 1.68E-11 |
| *AMOTL1* | 0.588 | 0.000154 | 0.000601 |
| *CAB39L* | 0.588 | 1.23E-22 | 7.15E-21 |
| *KNL1* | 0.587 | 1.55E-14 | 3.23E-13 |
| *CDCA8* | 0.584 | 5.57E-21 | 2.71E-19 |
| *CCDC159* | 0.581 | 5.06E-11 | 6.31E-10 |
| *KIF4A* | 0.581 | 7.69E-19 | 2.90E-17 |
| *MCM5* | 0.581 | 4.88E-25 | 3.86E-23 |
