# Supplementary material for: PACT is requisite for prostate cancer cell proliferation
Source: Sci Rep. 2025 Oct 21;15:36610. doi: 10.1038/s41598-025-20494-9 (PMC12540807; doi:10.1038/s41598-025-20494-9)
Supplement: Supplementary file 6 — Supplementary Material 6 [file 41598_2025_20494_MOESM6_ESM.docx]

**Supplementary Table 4.** Upregulated genes in the PACT KO cells as compared to parental LNCaP. The genes validated and further investigated herein are in bold and are namely: *NOVA1* (neuro-oncological ventral antigen-1), *PXDN* (Peroxidasin), and *RASSF2* (Ras association domain-containing protein 2), and were respectively upregulated 2.77, 2.33, and 1.4 log_2_ fold change, in the PACT KO cells

| **Gene Name** | **Log_2_ FC** | **P Value** | **FDR** |
| --- | --- | --- | --- |
| *NRXN1* | 3.858 | 5.48E-28 | 6.19E-26 |
| *MAGEC2* | 3.088 | 2.04E-26 | 1.92E-24 |
| *VCX* | 2.952 | 3.50E-24 | 2.46E-22 |
| ***NOVA1*** | **2.769** | **7.91E-48** | **9.38E-45** |
| *CBLN2* | 2.603 | 2.57E-49 | 3.66E-46 |
| *PHLDB2* | 2.505 | 2.85E-17 | 8.60E-16 |
| *GNG4* | 2.404 | 1.13E-30 | 1.76E-28 |
| ***PXDN*** | **2.332** | **4.18E-64** | **2.98E-60** |
| *LRRTM4* | 2.280 | 3.42E-08 | 2.72E-07 |
| *RSPO4* | 2.259 | 5.21E-06 | 2.80E-05 |
| *KCND2* | 2.242 | 2.58E-15 | 5.90E-14 |
| *ALDH1L1* | 2.197 | 3.23E-05 | 0.000146 |
| *ST6GALNAC5* | 2.195 | 1.73E-08 | 1.45E-07 |
| *KCNC2* | 2.116 | 5.65E-10 | 6.11E-09 |
| *UNC13C* | 2.064 | 3.85E-24 | 2.69E-22 |
| *VCX3A* | 2.036 | 0.000267 | 0.000986 |
| *MCAM* | 2.029 | 5.58E-12 | 8.08E-11 |
| *OR13C2* | 2.027 | 1.81E-05 | 8.63E-05 |
| *XDH* | 2.024 | 6.04E-14 | 1.16E-12 |
| *RAI2* | 2.011 | 4.90E-12 | 7.17E-11 |
| *ST8SIA4* | 1.998 | 9.47E-15 | 2.03E-13 |
| *VIM* | 1.992 | 7.51E-40 | 3.57E-37 |
| *TCF7L1* | 1.981 | 4.27E-07 | 2.81E-06 |
| *EPB41L4A* | 1.976 | 1.68E-19 | 6.83E-18 |
| *PAGE1* | 1.972 | 2.19E-24 | 1.57E-22 |
| *TENM2* | 1.950 | 5.77E-24 | 3.86E-22 |
| *FOXJ1* | 1.948 | 2.43E-08 | 1.98E-07 |
| *RFX6* | 1.944 | 5.37E-24 | 3.63E-22 |
| *CFAP126* | 1.922 | 3.54E-05 | 0.000158 |
| *COL5A1* | 1.875 | 5.54E-15 | 1.21E-13 |
| *PCDH15* | 1.830 | 4.86E-12 | 7.11E-11 |
| *ATP10D* | 1.830 | 5.75E-12 | 8.28E-11 |
| *CYP26B1* | 1.789 | 1.17E-06 | 7.05E-06 |
| *CSRNP3* | 1.732 | 8.23E-22 | 4.33E-20 |
| *WDR17* | 1.723 | 0.00032 | 0.001159 |
| *TUBB1* | 1.697 | 0.000446 | 0.00157 |
| *AC055839.2* | 1.693 | 0.00041 | 0.001456 |
| *PGM5* | 1.686 | 3.10E-05 | 0.00014 |
| *TNS1* | 1.666 | 4.42E-11 | 5.56E-10 |
| *ELOVL4* | 1.656 | 6.31E-08 | 4.79E-07 |
| *PLXNA4* | 1.643 | 2.42E-06 | 1.38E-05 |
| *PLXND1* | 1.640 | 6.36E-15 | 1.38E-13 |
| *ALB* | 1.617 | 4.70E-05 | 0.000205 |
| *NLGN4X* | 1.541 | 8.26E-23 | 4.92E-21 |
| *CYP2S1* | 1.535 | 6.22E-13 | 1.03E-11 |
| *ESRRG* | 1.521 | 3.83E-12 | 5.70E-11 |
| *FBXL2* | 1.517 | 4.39E-05 | 0.000193 |
| *IRX6* | 1.480 | 4.79E-05 | 0.000209 |
| *SPEG* | 1.467 | 3.26E-05 | 0.000147 |
| *ZNF619* | 1.463 | 4.71E-08 | 3.66E-07 |
| *RCAN2* | 1.442 | 3.55E-18 | 1.20E-16 |
| *CDHR1* | 1.440 | 3.62E-12 | 5.41E-11 |
| *MS4A8* | 1.425 | 5.34E-13 | 8.95E-12 |
| *ZNF454* | 1.416 | 4.37E-07 | 2.87E-06 |
| *ITGB7* | 1.415 | 3.22E-14 | 6.43E-13 |
| ***RASSF2*** | **1.396** | **1.04E-31** | **1.79E-29** |
| *ARMCX4* | 1.378 | 1.61E-10 | 1.88E-09 |
| *FSIP2* | 1.362 | 2.56E-05 | 0.000118 |
| *DCDC2* | 1.362 | 5.46E-06 | 2.92E-05 |
| *SOCS3* | 1.350 | 6.55E-06 | 3.44E-05 |
| *FBLN1* | 1.348 | 6.54E-06 | 3.44E-05 |
| *CCN3* | 1.346 | 4.89E-10 | 5.36E-09 |
| *SLC15A2* | 1.344 | 1.19E-09 | 1.23E-08 |
| *ACOX2* | 1.338 | 2.56E-11 | 3.36E-10 |
| *SP8* | 1.326 | 0.000368 | 0.001322 |
| *FGFR2* | 1.324 | 1.87E-12 | 2.90E-11 |
| *COL4A4* | 1.320 | 5.38E-05 | 0.000232 |
| *PTPRB* | 1.314 | 1.29E-13 | 2.36E-12 |
| *FAM110B* | 1.312 | 2.73E-17 | 8.30E-16 |
| *PDLIM3* | 1.311 | 0.001432 | 0.004461 |
| *ACE2* | 1.287 | 8.90E-06 | 4.56E-05 |
| *CDH18* | 1.271 | 2.38E-07 | 1.65E-06 |
| *ADGRL2* | 1.268 | 0.002648 | 0.00766 |
| *SAMD5* | 1.240 | 1.19E-18 | 4.35E-17 |
| *RALYL* | 1.238 | 3.16E-29 | 4.13E-27 |
| *MSI1* | 1.233 | 2.67E-20 | 1.19E-18 |
| *RAG1* | 1.233 | 0.002962 | 0.008452 |
| *TSHZ2* | 1.228 | 1.47E-07 | 1.05E-06 |
| *CALCRL* | 1.226 | 4.39E-15 | 9.72E-14 |
| *CDKN1C* | 1.189 | 1.38E-08 | 1.17E-07 |
| *NSG1* | 1.184 | 3.07E-13 | 5.37E-12 |
| *PDZD4* | 1.173 | 1.13E-21 | 5.89E-20 |
| *PRRT4* | 1.171 | 0.002082 | 0.006204 |
| *NCALD* | 1.169 | 8.42E-05 | 0.000349 |
| *HOXC8* | 1.161 | 2.91E-07 | 1.98E-06 |
| *SNAP91* | 1.155 | 1.76E-11 | 2.37E-10 |
| *CAPS2* | 1.152 | 1.44E-20 | 6.70E-19 |
| *ERAP2* | 1.137 | 1.73E-06 | 1.02E-05 |
| *NTF4* | 1.137 | 0.000881 | 0.002891 |
| *NRXN3* | 1.135 | 0.001715 | 0.005237 |
| *SI* | 1.128 | 7.82E-33 | 1.48E-30 |
| *HEPH* | 1.117 | 1.45E-07 | 1.04E-06 |
| *RDH10* | 1.116 | 3.52E-42 | 2.39E-39 |
| *RBPMS2* | 1.114 | 5.27E-13 | 8.84E-12 |
| *ZNF737* | 1.110 | 5.39E-32 | 9.59E-30 |
| *CHRNA3* | 1.106 | 2.17E-08 | 1.79E-07 |
| *HOXA10* | 1.100 | 7.20E-16 | 1.78E-14 |
| *FRMD3* | 1.098 | 3.55E-10 | 3.96E-09 |
| *BEAN1* | 1.088 | 0.003575 | 0.00995 |
| *DRC7* | 1.087 | 0.000992 | 0.003219 |
| *MAGEA1* | 1.085 | 7.16E-16 | 1.78E-14 |
| *AFAP1L2* | 1.079 | 8.17E-06 | 4.21E-05 |
| *CD24* | 1.070 | 1.44E-42 | 1.08E-39 |
| *CNTN3* | 1.070 | 0.000223 | 0.000841 |
| *ID3* | 1.066 | 1.32E-10 | 1.56E-09 |
| *RIMS4* | 1.065 | 8.65E-23 | 5.07E-21 |
| *GLIPR1* | 1.065 | 7.95E-05 | 0.000332 |
| *MEST* | 1.057 | 3.86E-34 | 8.73E-32 |
| *SCN1B* | 1.050 | 0.00097 | 0.003154 |
| *CYP2E1* | 1.048 | 0.000142 | 0.00056 |
| *TMEM232* | 1.047 | 5.81E-05 | 0.000249 |
| *PARP14* | 1.044 | 1.12E-14 | 2.36E-13 |
| *CAMK2B* | 1.042 | 5.40E-22 | 2.89E-20 |
| *POTEF* | 1.040 | 0.003365 | 0.009443 |
| *CACNA1G* | 1.037 | 3.93E-09 | 3.65E-08 |
| *UCP2* | 1.037 | 4.96E-05 | 0.000215 |
| *ADRA1A* | 1.024 | 4.59E-12 | 6.75E-11 |
| *STON2* | 1.021 | 2.54E-31 | 4.20E-29 |
| *DUOX2* | 1.020 | 0.000291 | 0.001066 |
| *RASSF6* | 1.013 | 0.000954 | 0.003106 |
| *AMH* | 1.010 | 1.55E-05 | 7.52E-05 |
| *EPAS1* | 1.003 | 4.17E-08 | 3.26E-07 |
| *EFNB3* | 0.993 | 2.78E-19 | 1.10E-17 |
| *GSDME* | 0.992 | 3.04E-16 | 7.91E-15 |
| *RAB27A* | 0.989 | 1.38E-26 | 1.37E-24 |
| *ACSM1* | 0.984 | 5.97E-05 | 0.000255 |
| *SDK1* | 0.982 | 4.64E-10 | 5.09E-09 |
| *RAPGEF4* | 0.981 | 1.23E-05 | 6.10E-05 |
| *NMNAT2* | 0.980 | 3.40E-27 | 3.55E-25 |
| *SCD5* | 0.978 | 1.42E-26 | 1.40E-24 |
| *NR4A2* | 0.977 | 6.25E-05 | 0.000266 |
| *ZNF648* | 0.973 | 4.71E-15 | 1.04E-13 |
| *ESAM* | 0.967 | 0.000392 | 0.001401 |
| *KLF8* | 0.960 | 1.20E-08 | 1.03E-07 |
| *MAP2* | 0.960 | 5.97E-29 | 7.59E-27 |
| *EPS8L1* | 0.959 | 0.000466 | 0.001636 |
| *CTAGE4* | 0.957 | 0.000768 | 0.002552 |
| *SLC38A3* | 0.955 | 9.65E-07 | 5.92E-06 |
| *NXN* | 0.949 | 9.14E-37 | 2.96E-34 |
| *KLHL32* | 0.946 | 0.000512 | 0.001777 |
| *VAMP5* | 0.941 | 0.002871 | 0.008219 |
| *ATP11A* | 0.941 | 4.73E-53 | 9.63E-50 |
| *HTRA1* | 0.940 | 1.00E-12 | 1.62E-11 |
| *SPATA1* | 0.938 | 0.001166 | 0.003719 |
| *TNIK* | 0.929 | 1.61E-12 | 2.51E-11 |
| *BMP6* | 0.928 | 2.38E-16 | 6.38E-15 |
| *TMEM163* | 0.925 | 2.61E-05 | 0.00012 |
| *CACNA1H* | 0.925 | 4.99E-06 | 2.69E-05 |
| *SYT3* | 0.923 | 0.000503 | 0.001751 |
| *RADX* | 0.919 | 3.69E-05 | 0.000164 |
| *NRG4* | 0.913 | 5.58E-07 | 3.60E-06 |
| *ZNF253* | 0.908 | 1.16E-26 | 1.16E-24 |
| *PRIMA1* | 0.905 | 0.000545 | 0.001876 |
| *SERPINB5* | 0.902 | 1.76E-31 | 2.98E-29 |
| *SRSF12* | 0.900 | 0.000109 | 0.000442 |
| *BCL11B* | 0.900 | 0.001369 | 0.004285 |
| *CACNA2D2* | 0.892 | 7.78E-23 | 4.65E-21 |
| *SH3RF3* | 0.890 | 6.91E-07 | 4.38E-06 |
| *IL1RAPL1* | 0.883 | 3.55E-08 | 2.81E-07 |
| *PTGES3L* | 0.880 | 0.003587 | 0.009973 |
| *ENPP5* | 0.879 | 1.63E-35 | 4.54E-33 |
| *TMEM59L* | 0.875 | 1.33E-22 | 7.64E-21 |
| *MCTP1* | 0.874 | 2.94E-14 | 5.88E-13 |
| *STK32A* | 0.873 | 4.10E-11 | 5.20E-10 |
| *MANEA* | 0.872 | 2.60E-50 | 4.11E-47 |
| *CACNB2* | 0.869 | 1.83E-08 | 1.52E-07 |
| *PDGFA* | 0.867 | 8.38E-09 | 7.31E-08 |
| *CAPN5* | 0.862 | 1.19E-28 | 1.45E-26 |
| *AZIN2* | 0.861 | 1.89E-07 | 1.33E-06 |
| *EFEMP2* | 0.857 | 9.05E-07 | 5.59E-06 |
| *GPX8* | 0.852 | 1.73E-20 | 8.04E-19 |
| *NAALAD2* | 0.852 | 9.62E-11 | 1.16E-09 |
| *IFI27L2* | 0.851 | 1.67E-09 | 1.66E-08 |
| *PLCL2* | 0.851 | 0.000676 | 0.002277 |
| *FAM189A2* | 0.850 | 0.002684 | 0.007741 |
| *CYP3A5* | 0.849 | 5.66E-07 | 3.65E-06 |
| *ZCCHC18* | 0.845 | 0.00209 | 0.006224 |
| *ERO1B* | 0.843 | 2.43E-16 | 6.48E-15 |
| *TUBA1A* | 0.842 | 8.46E-42 | 5.02E-39 |
| *DDX60L* | 0.837 | 0.000486 | 0.001699 |
| *RNF165* | 0.837 | 1.52E-08 | 1.28E-07 |
| *SLC22A31* | 0.830 | 4.80E-15 | 1.06E-13 |
| *ATP6V1FNB* | 0.830 | 2.41E-09 | 2.32E-08 |
| *MID1* | 0.825 | 3.40E-24 | 2.39E-22 |
| *SLC6A16* | 0.824 | 1.46E-12 | 2.29E-11 |
| *EFHB* | 0.819 | 0.002677 | 0.007727 |
| *WLS* | 0.819 | 2.01E-26 | 1.92E-24 |
| *VWA2* | 0.818 | 4.01E-25 | 3.20E-23 |
| *LRP1* | 0.813 | 2.26E-38 | 8.45E-36 |
| *SAMD14* | 0.813 | 0.002335 | 0.006866 |
| *SOCS2* | 0.812 | 6.42E-14 | 1.23E-12 |
| *TIMP3* | 0.809 | 0.0006 | 0.002042 |
| *KCNIP3* | 0.808 | 0.001896 | 0.00573 |
| *VAMP7* | 0.798 | 2.45E-38 | 8.94E-36 |
| *RASGRP1* | 0.798 | 0.000198 | 0.000754 |
| *BACE2* | 0.797 | 0.000557 | 0.001911 |
| *SPOCK1* | 0.796 | 8.28E-05 | 0.000343 |
| *BCHE* | 0.794 | 1.50E-41 | 8.57E-39 |
| *NLRP11* | 0.792 | 0.002949 | 0.008423 |
| *LTBP1* | 0.791 | 3.73E-10 | 4.15E-09 |
| *HOXB3* | 0.788 | 0.002885 | 0.008253 |
| *ANO7* | 0.788 | 5.76E-11 | 7.15E-10 |
| *MUC4* | 0.787 | 0.002219 | 0.006562 |
| *KCNH3* | 0.786 | 5.25E-07 | 3.41E-06 |
| *HSPA5* | 0.784 | 4.90E-43 | 4.10E-40 |
| *KALRN* | 0.783 | 8.27E-19 | 3.08E-17 |
| *KIAA0825* | 0.782 | 0.000192 | 0.000736 |
| *NANOS1* | 0.781 | 8.70E-08 | 6.45E-07 |
| *GPC6* | 0.777 | 4.43E-06 | 2.41E-05 |
| *HSP90B1* | 0.774 | 1.15E-47 | 1.26E-44 |
| *CHST15* | 0.773 | 1.27E-08 | 1.08E-07 |
| *DNAJB9* | 0.773 | 3.80E-18 | 1.29E-16 |
| *MTSS1* | 0.771 | 0.00024 | 0.000898 |
| *RIMS3* | 0.771 | 3.96E-14 | 7.79E-13 |
| *ZNF578* | 0.770 | 3.09E-09 | 2.92E-08 |
| *ZNF93* | 0.769 | 4.82E-09 | 4.39E-08 |
| *FAM177B* | 0.767 | 2.15E-05 | 0.000101 |
| *PLXDC2* | 0.767 | 6.64E-21 | 3.19E-19 |
| *CCDC30* | 0.767 | 0.000331 | 0.001197 |
| *SPEF1* | 0.766 | 0.000927 | 0.003029 |
| *SYT1* | 0.765 | 0.000509 | 0.001768 |
| *CAMK1D* | 0.764 | 8.91E-05 | 0.000367 |
| *SPAG16* | 0.762 | 4.47E-15 | 9.87E-14 |
| *TNS3* | 0.759 | 0.000911 | 0.002982 |
| *CITED2* | 0.758 | 1.25E-37 | 4.24E-35 |
| *KIF7* | 0.757 | 0.001719 | 0.005248 |
| *PTPRR* | 0.756 | 4.37E-16 | 1.11E-14 |
| *TSHZ3* | 0.755 | 2.13E-09 | 2.06E-08 |
| *ABCC8* | 0.755 | 5.92E-14 | 1.14E-12 |
| *FSD1L* | 0.751 | 3.66E-20 | 1.60E-18 |
| *BVES* | 0.750 | 4.18E-15 | 9.28E-14 |
| *SERPINE2* | 0.750 | 5.89E-12 | 8.48E-11 |
| *ADORA1* | 0.749 | 0.000757 | 0.002518 |
| *NIPSNAP3B* | 0.749 | 1.50E-08 | 1.27E-07 |
| *HES2* | 0.746 | 1.51E-08 | 1.28E-07 |
| *WWTR1* | 0.746 | 0.002159 | 0.006411 |
| *TMEM91* | 0.745 | 1.13E-05 | 5.63E-05 |
| *MAPK10* | 0.744 | 0.000179 | 0.00069 |
| *ROR2* | 0.740 | 0.000562 | 0.001926 |
| *GFPT2* | 0.739 | 1.09E-05 | 5.47E-05 |
| *SAT1* | 0.736 | 6.06E-35 | 1.49E-32 |
| *HHAT* | 0.732 | 5.93E-08 | 4.52E-07 |
| *MAN1A1* | 0.728 | 3.57E-15 | 8.01E-14 |
| *IGIP* | 0.728 | 1.12E-15 | 2.72E-14 |
| *KCNMB4* | 0.728 | 0.000783 | 0.002595 |
| *TMEM158* | 0.726 | 4.91E-06 | 2.65E-05 |
| *CTTNBP2* | 0.726 | 0.002228 | 0.006586 |
| *EBF3* | 0.722 | 1.63E-09 | 1.64E-08 |
| *TNFRSF25* | 0.722 | 0.000998 | 0.003232 |
| *BLNK* | 0.721 | 3.97E-14 | 7.80E-13 |
| *PIGZ* | 0.718 | 3.94E-12 | 5.84E-11 |
| *MAPK4* | 0.715 | 4.05E-05 | 0.000179 |
| *TTC16* | 0.713 | 0.000318 | 0.001154 |
| *STAT6* | 0.713 | 0.000116 | 0.000467 |
| *DDX60* | 0.711 | 1.34E-07 | 9.73E-07 |
| *C1R* | 0.711 | 6.32E-30 | 8.74E-28 |
| *KCNN2* | 0.711 | 2.82E-30 | 4.06E-28 |
| *NFASC* | 0.711 | 5.58E-08 | 4.27E-07 |
| *FYN* | 0.711 | 2.43E-05 | 0.000113 |
| *PALMD* | 0.710 | 1.80E-09 | 1.78E-08 |
| *OPTN* | 0.709 | 7.47E-18 | 2.44E-16 |
| *DSEL* | 0.706 | 3.00E-39 | 1.25E-36 |
| *LDLRAD1* | 0.706 | 0.002027 | 0.006059 |
| *ARNT2* | 0.705 | 2.18E-31 | 3.66E-29 |
| *SERPINI1* | 0.704 | 1.25E-23 | 8.18E-22 |
| *NRG3* | 0.701 | 0.001673 | 0.005123 |
| *CPQ* | 0.699 | 4.44E-14 | 8.67E-13 |
| *PTPRK* | 0.698 | 2.72E-16 | 7.16E-15 |
| *EMID1* | 0.697 | 1.77E-06 | 1.04E-05 |
| *NFATC4* | 0.696 | 5.45E-08 | 4.18E-07 |
| *TMEM266* | 0.696 | 3.44E-06 | 1.91E-05 |
| *TMEFF2* | 0.695 | 1.81E-39 | 7.80E-37 |
| *TSPAN1* | 0.695 | 5.24E-26 | 4.64E-24 |
| *ZNF595* | 0.693 | 1.82E-19 | 7.38E-18 |
| *ZC4H2* | 0.692 | 5.17E-14 | 1.00E-12 |
| *PNMA2* | 0.692 | 6.54E-09 | 5.81E-08 |
| *PHLDA1* | 0.691 | 8.82E-23 | 5.14E-21 |
| *CD274* | 0.691 | 1.82E-09 | 1.80E-08 |
| *RLN2* | 0.691 | 4.47E-10 | 4.93E-09 |
| *PAQR8* | 0.690 | 7.92E-07 | 4.95E-06 |
| *PDGFRL* | 0.689 | 2.27E-06 | 1.30E-05 |
| *PGM2L1* | 0.687 | 5.52E-11 | 6.87E-10 |
| *ADAM23* | 0.686 | 1.32E-09 | 1.35E-08 |
| *MANF* | 0.685 | 9.97E-32 | 1.73E-29 |
| *EFCC1* | 0.683 | 1.38E-06 | 8.22E-06 |
| *WDR66* | 0.683 | 2.39E-09 | 2.30E-08 |
| *ZNF491* | 0.683 | 0.000225 | 0.000846 |
| *TMEM170A* | 0.678 | 1.38E-10 | 1.63E-09 |
| *SMOC2* | 0.678 | 7.90E-08 | 5.89E-07 |
| *GNAQ* | 0.677 | 1.22E-20 | 5.72E-19 |
| *RFX2* | 0.676 | 0.000435 | 0.001537 |
| *SLC25A29* | 0.675 | 8.34E-39 | 3.39E-36 |
| *SLC44A5* | 0.675 | 0.001248 | 0.003952 |
| *PLEKHB1* | 0.673 | 1.23E-42 | 9.72E-40 |
| *MAP3K12* | 0.673 | 3.97E-19 | 1.54E-17 |
| *GLIPR1L2* | 0.672 | 0.001926 | 0.005809 |
| *SLC1A1* | 0.667 | 2.47E-18 | 8.57E-17 |
| *PDZRN3* | 0.666 | 2.11E-22 | 1.19E-20 |
| *FBLN2* | 0.665 | 5.30E-12 | 7.71E-11 |
| *TRIM2* | 0.662 | 4.51E-06 | 2.45E-05 |
| *TVP23C* | 0.662 | 0.000206 | 0.000782 |
| *SLC4A4* | 0.660 | 4.03E-28 | 4.63E-26 |
| *SYNPO* | 0.659 | 0.000312 | 0.001136 |
| *TENM1* | 0.658 | 1.57E-06 | 9.28E-06 |
| *PELI2* | 0.656 | 1.42E-11 | 1.93E-10 |
| *PLCG2* | 0.655 | 2.86E-05 | 0.00013 |
| *PLCB2* | 0.653 | 0.000125 | 0.000501 |
| *IKZF2* | 0.653 | 5.64E-09 | 5.07E-08 |
| *GPR37* | 0.652 | 7.79E-05 | 0.000326 |
| *ITGB2* | 0.652 | 0.000584 | 0.001995 |
| *FAHD2B* | 0.651 | 8.14E-10 | 8.59E-09 |
| *RNASE4* | 0.649 | 1.56E-07 | 1.11E-06 |
| *SULF2* | 0.648 | 0.001131 | 0.003622 |
| *SEL1L* | 0.647 | 1.52E-40 | 8.00E-38 |
| *CHST11* | 0.645 | 9.26E-07 | 5.70E-06 |
| *GLIS2* | 0.644 | 1.38E-11 | 1.88E-10 |
| *METRNL* | 0.643 | 1.92E-08 | 1.59E-07 |
| *TINAGL1* | 0.643 | 0.00092 | 0.003009 |
| *VWDE* | 0.641 | 0.000688 | 0.002311 |
| *SPTBN1* | 0.639 | 5.38E-07 | 3.49E-06 |
| *ZSWIM6* | 0.638 | 1.11E-14 | 2.35E-13 |
| *COL27A1* | 0.637 | 1.29E-05 | 6.36E-05 |
| *EPHA7* | 0.637 | 1.61E-13 | 2.93E-12 |
| *IFIT2* | 0.635 | 9.27E-07 | 5.70E-06 |
| *FBXL7* | 0.634 | 1.85E-09 | 1.83E-08 |
| *ST6GALNAC1* | 0.632 | 2.57E-06 | 1.46E-05 |
| *BOC* | 0.632 | 2.70E-06 | 1.52E-05 |
| *NREP* | 0.628 | 6.84E-34 | 1.44E-31 |
| *ACSL4* | 0.627 | 3.11E-11 | 4.01E-10 |
| *ZBTB20* | 0.624 | 5.72E-14 | 1.11E-12 |
| *PCP2* | 0.623 | 0.001337 | 0.004201 |
| *MINDY1* | 0.622 | 9.47E-11 | 1.14E-09 |
| *ARHGEF3* | 0.622 | 4.21E-05 | 0.000186 |
| *TCEAL3* | 0.621 | 1.70E-14 | 3.52E-13 |
| *NFATC2* | 0.618 | 1.06E-05 | 5.37E-05 |
| *VPS37D* | 0.618 | 4.57E-05 | 0.0002 |
| *RAB6C* | 0.618 | 4.18E-10 | 4.62E-09 |
| *CYB5R2* | 0.617 | 4.35E-07 | 2.86E-06 |
| *HSD17B14* | 0.617 | 1.24E-06 | 7.45E-06 |
| *MYOZ1* | 0.613 | 4.42E-07 | 2.90E-06 |
| *GSTA1* | 0.612 | 2.53E-13 | 4.48E-12 |
| *BRSK1* | 0.611 | 0.00042 | 0.001489 |
| *RNF150* | 0.611 | 1.64E-09 | 1.64E-08 |
| *ANKDD1A* | 0.610 | 0.000387 | 0.001383 |
| *CENPV* | 0.610 | 1.53E-09 | 1.54E-08 |
| *MKRN2OS* | 0.610 | 5.03E-06 | 2.71E-05 |
| *BDH2* | 0.607 | 3.33E-10 | 3.73E-09 |
| *PITPNM1* | 0.607 | 1.87E-21 | 9.46E-20 |
| *KCNRG* | 0.606 | 7.95E-05 | 0.000332 |
| *NR6A1* | 0.604 | 1.91E-08 | 1.59E-07 |
| *ADAP2* | 0.603 | 2.65E-05 | 0.000122 |
| *RAB6D* | 0.603 | 2.36E-05 | 0.00011 |
| *FRMPD2* | 0.601 | 2.87E-14 | 5.75E-13 |
| *ZFP2* | 0.600 | 0.000591 | 0.002014 |
| *MLLT3* | 0.600 | 7.81E-17 | 2.20E-15 |
| *CRYM* | 0.599 | 1.01E-06 | 6.17E-06 |
| *SPEF2* | 0.599 | 1.62E-06 | 9.54E-06 |
| *DNAJC28* | 0.599 | 1.07E-05 | 5.39E-05 |
| *PALM* | 0.599 | 5.12E-07 | 3.33E-06 |
| *PPP4R4* | 0.597 | 0.001982 | 0.00595 |
| *SRGAP1* | 0.597 | 1.83E-22 | 1.04E-20 |
| *MYO1B* | 0.593 | 2.56E-11 | 3.35E-10 |
| *ATP8A1* | 0.593 | 4.81E-13 | 8.14E-12 |
| *KIAA1211L* | 0.592 | 0.000805 | 0.00266 |
| *USP2* | 0.590 | 2.61E-07 | 1.79E-06 |
| *LCN12* | 0.587 | 0.000316 | 0.001148 |
| *PLXNA3* | 0.586 | 3.71E-23 | 2.29E-21 |
| *DYRK3* | 0.586 | 1.46E-05 | 7.10E-05 |
| *IGF2BP3* | 0.585 | 5.99E-09 | 5.36E-08 |
| *SPTB* | 0.585 | 0.000214 | 0.000809 |
| *NUCB2* | 0.585 | 3.63E-38 | 1.29E-35 |
| *GPR161* | 0.582 | 2.36E-20 | 1.07E-18 |
| *IRS2* | 0.581 | 0.000978 | 0.003174 |
| *DDX58* | 0.581 | 1.13E-11 | 1.56E-10 |
| *CLIP3* | 0.580 | 1.31E-05 | 6.44E-05 |
